# Zirconium Metal–Organic Polyhedra with Dual Behavior for Organophosphate Poisoning Treatment

**DOI:** 10.1021/acsami.2c06025

**Published:** 2022-06-02

**Authors:** Pedro Delgado, Javier D. Martin-Romera, Cristina Perona, Rebecca Vismara, Simona Galli, Carmen R. Maldonado, Francisco J. Carmona, Natalia M. Padial, Jorge A. R. Navarro

**Affiliations:** † Departamento de Química Inorgánica, Universidad de Granada, Av. Fuentenueva S/N, 18071 Granada, Spain; ‡ Dipartimento di Scienza e Alta Tecnologia, Università degli Studi dell‘Insubria, Via Valleggio 11, 22100 Como, Italy; § Functional Inorganic Materials Team, Instituto de Ciencia Molecular (ICMol), Universitat de València, 46980 València, Spain

**Keywords:** nerve agents, host−guest chemistry, pesticide, controlled drug delivery, metal−organic cages

## Abstract

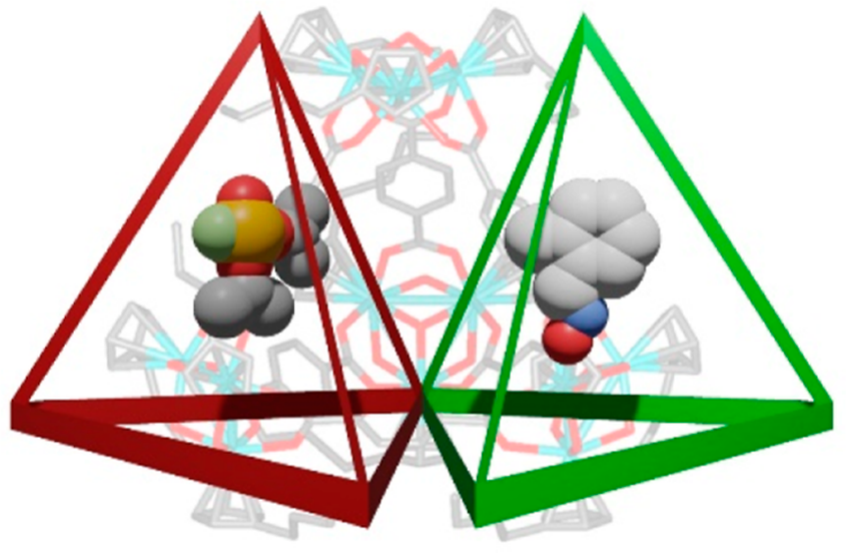

Organophosphate nerve agents and pesticides are extremely toxic compounds because they result in acetylcholinesterase (AChE) inhibition and concomitant nerve system damage. Herein, we report the synthesis, structural characterization, and proof-of-concept utility of zirconium metal–organic polyhedra (Zr-MOPs) for organophosphate poisoning treatment. The results show the formation of robust tetrahedral cages [((*n*-butylCpZr)_3_(OH)_3_O)_4_L_6_]Cl_6_ (**Zr-MOP-1**; L = benzene-1,4-dicarboxylate, *n*-butylCp = *n*-butylcyclopentadienyl, **Zr-MOP-10**, and L = 4,4′-biphenyldicarboxylate) decorated with lipophilic alkyl residues and possessing accessible cavities of ∼9.8 and ∼10.7 Å inner diameters, respectively. These systems are able to both capture the organophosphate model compound diisopropylfluorophosphate (DIFP) and host and release the AChE reactivator drug pralidoxime (2-PAM). The resulting **2-PAM@Zr-MOP-1(0)** host–guest assemblies feature a sustained delivery of 2-PAM under simulated biological conditions, with a concomitant reactivation of DIFP-inhibited AChE. Finally, **2-PAM@Zr-MOP** systems have been incorporated into biocompatible phosphatidylcholine liposomes with the resulting assemblies being non-neurotoxic, as proven using neuroblastoma cell viability assays.

## Introduction

One of the greatest challenges of the 21st century is to increase the production of crops in order to reach the high demand of food of the ever increasing global population (from the current 7.6 billion to the 10 billion expected by 2050).^1^ This challenge involves extensive use of pesticides, which has the side effect of posing a real threat to the human health (110,000 deaths/year and 5 million pesticide-related illnesses), aquatic ecosystems, and the environment at large.^2,3^ Organophosphorous-based pesticides and chemical warfare nerve agents are extremely toxic compounds as a consequence of their easy penetration through biological tissues and consequent damage of the central nervous system due to irreversible inhibition of acetylcholinesterase (AChE) activity. Reactivation of inhibited AChE using oximes, such as pralidoxime (2-PAM), is the treatment of choice for organophosphate poisoning.^4^ AChE reactivators are able to remove the phosphonate moiety from the serine active site, restoring the activity of the enzyme. However, in order to become effective, oxime treatment needs to be continuous over time.^5,6^ On top of that, poor drug permeation through the blood–brain barrier needs to be improved. Consequently, the development of new materials suitable as drug vehicles/delivery systems is of high interest.

Zirconium metal–organic frameworks (Zr-MOFs) based on [Zr_6_O_4_(OH)_4_] secondary building units are being thoroughly studied for organophosphate capture and hydrolytic degradation due to the suitable combination of material robustness, high pore accessibility, and highly Lewis acidic zirconium metal centers.^7−10^ However, the extended nature of these materials, unless in the nanometric form, make them unsuitable for organophosphate poisoning treatment. In this regard, metal–organic polyhedra (MOPs),^11−13^ which can be considered downsized units of MOFs, are characterized by both a rich solid chemistry and a rich solution chemistry. The MOP solution processability can lead to increased biocompatibility, paving the way to their use as vehicles of bioactive molecules. Indeed, recent results by Liu and colleagues show the ability of chiral zirconium-MOPs@liposome assemblies to selectively transport amino acids through cell membranes.^14^

**Scheme 1:**
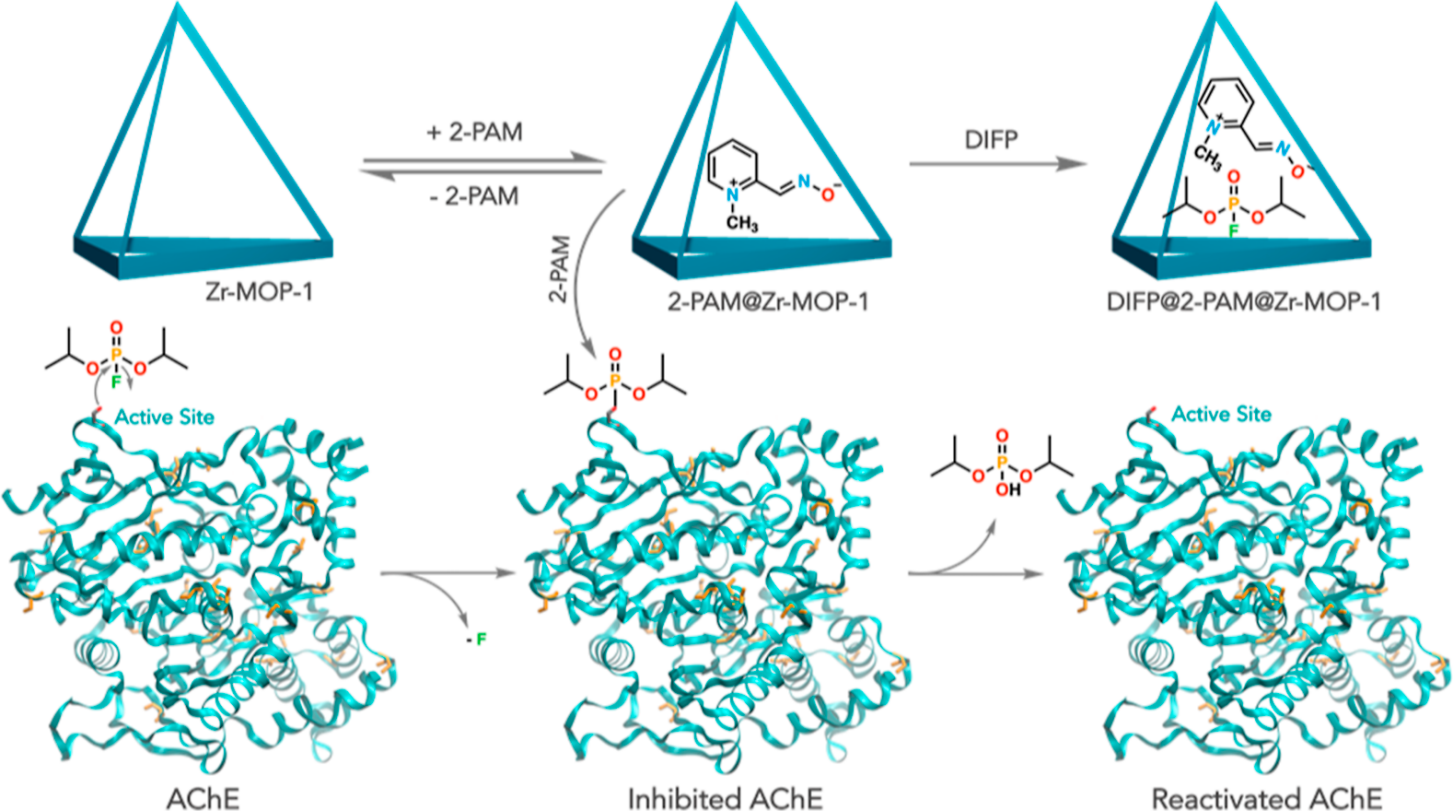
Summary of the Dual Behavior of Zr-MOP Assemblies for Nerve Agent Simulant DIFP Capture, 2-PAM Drug Controlled Release, and AChE^15^ Reactivation

In this work, we hypothesize that zirconium-MOPs, with appropriate functionalization, may transport/release drug molecules via cell internalization and/or diffusion through the hematoencephalic junction, allowing the development of new platforms for organophosphate intoxication treatment. With this aim, we report the formation of robust non-neurotoxic *n*-butyl decorated tetrahedral zirconium MOPs (Zr-MOPs). These cage assemblies behave as dual materials for organophosphate intoxication treatment, being able to capture the nerve agent simulant diisopropylfluorophosphate (DIFP) and to host and release the 2-PAM drug under simulated physiological conditions, with a concomitant reactivation of DIFP-inhibited AChE (Scheme 1).

## Results and Discussion

### Synthesis and Structural Characterization

**Figure 1:**
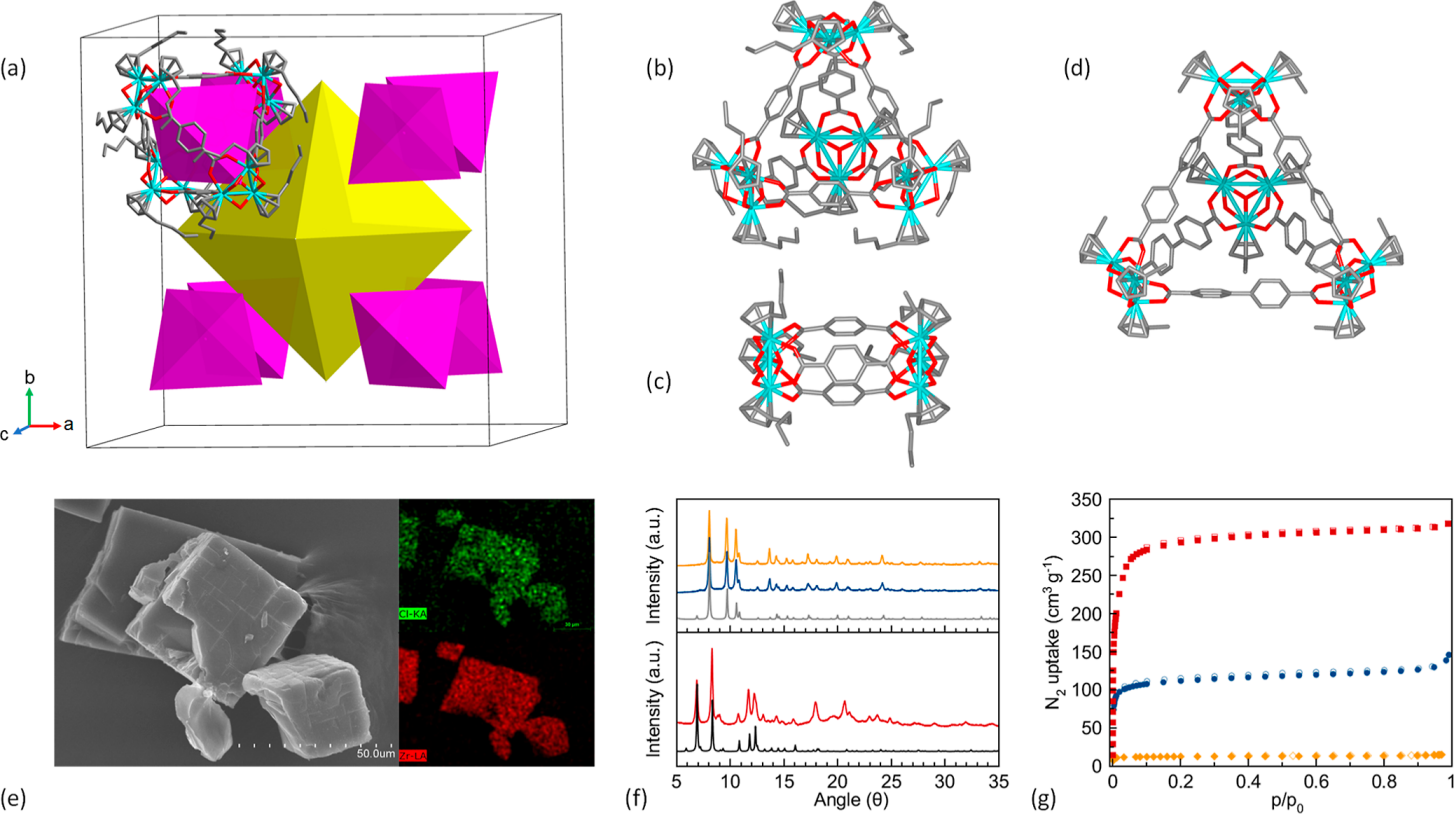
(a) Simplified crystal structure of **Zr-MOP-1-NH**_**2**_: the fuchsia tetrahedra stand for the tetrameric cages and the yellow octahedron highlights the central cavity. (b) Tetrahedral cage of **Zr-MOP-1-NH**_**2**_. (c) Dimeric isomer **Zr-MOP-1′**. (d) Tetrahedral cage of **Zr-MOP-10**, isoreticular to **Zr-MOP-1-NH**_**2**_. Color code: zirconium, light blue; carbon, gray; and oxygen, red. Hydrogens and disordered NH_2_ residues are not depicted for clarity. (e) SEM–EDX images of Zr-MOP-1; color code: Cl, green and Zr, red. (f) On the top: comparison between the observed PXRD patterns of **Zr-MOP-1** (blue trace) and **Zr-MOP-1-NH**_**2**_ (yellow trace) and the simulated PXRD pattern of **Zr-MOP-1-NH**_**2**_ (gray trace). On the bottom: experimental Zr-MOP-10 (red trace) and simulated **Zr-MOP-10** PXRD patterns (black trace). (g) N_2_ adsorption isotherms at 77 K for **Zr-MOP-1-X** (X = H and NH_2_, blue circles and yellow diamonds, respectively) and **Zr-MOP-10** (red squares). Open symbols denote desorption.

**Figure 2:**
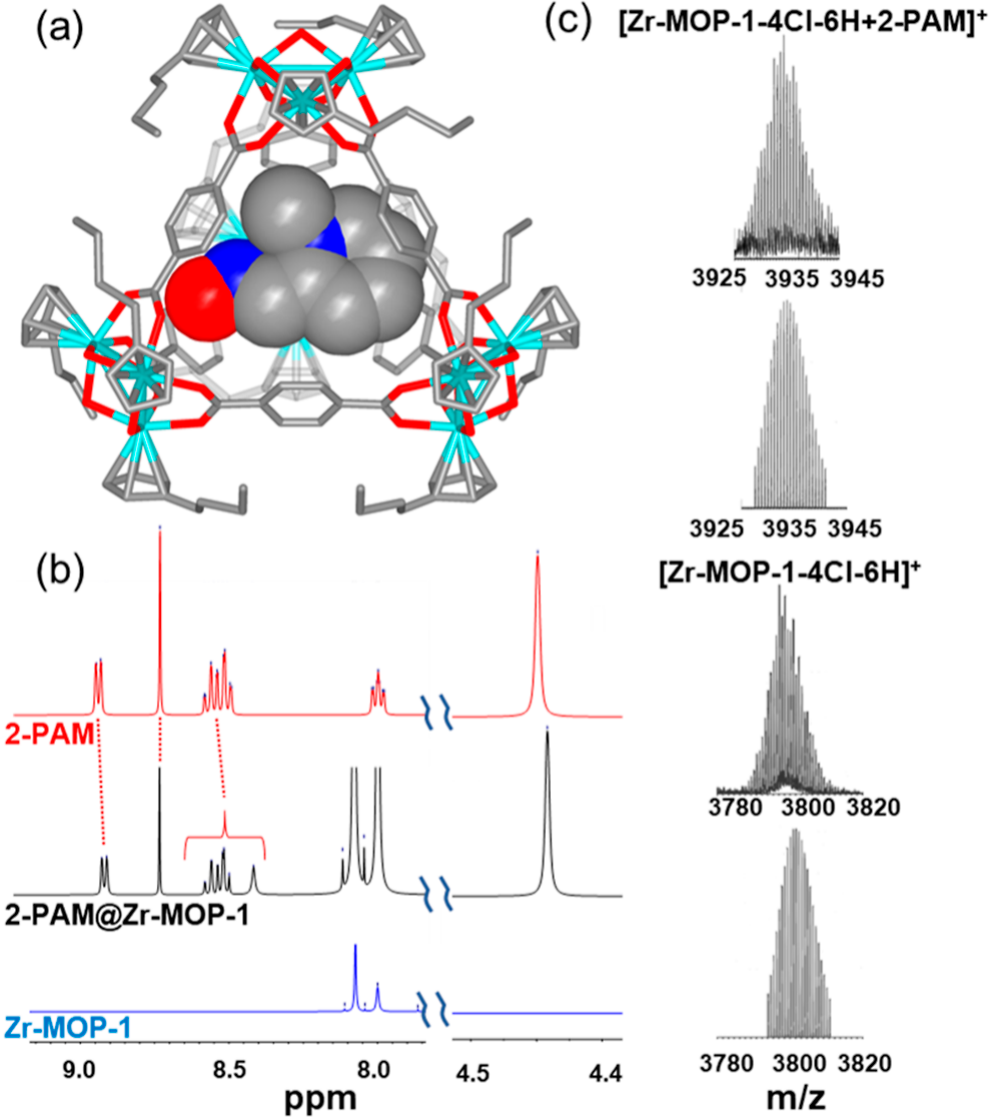
(a) Computational model of 2-PAM encapsulated in Zr-MOP-1 cages; (b) ^1^H NMR spectra of free Zr-MOP-1, 2-PAM, and the 2-PAM@Zr-MOP-1 host–guest assembly; (c) ESI-MS spectra of Zr-MOP-1 and 2-PAM@Zr-MOP-1.

The partial hydrolysis of (*n*-butylCp)_2_ZrCl_2_ in wet dimethylformamide (DMF) and the consecutive coordination to carboxylate organic linkers at 70 °C lead to the formation of robust *n*-butyl decorated tetrahedral Zr-MOPs of [((*n*-butylCpZr)_3_(OH)_3_O)_4_L_6_]Cl_6_ formulation (**Zr-MOP-1**, L = benzene-1,4-dicarboxylate and *n*-butylCp = *n*-butylcyclopentadienyl; **Zr-MOP-1-NH**_**2**_, L = benzene-1,4-dicarboxylate-2-amino; and **Zr-MOP-10**, L = 4,4′-biphenyldicarboxylate), as established using ^1^H NMR, electrospray ionization mass spectrometry (ESI-MS), scanning electron microscopy (SEM)–energy-dispersive X-ray spectroscopy (EDX), and powder and single-crystal X-ray crystallography (Figures 1, 2, S1–S3, S9–S16, and S21–S24). Further heating of the reaction mixture to 80 °C leads to the formation of dimeric isomers of a cigar-like shape of [((*n*-butylCpZr)_3_(OH)_3_O)_2_L_3_]Cl_2_ formulation, which we denote as **Zr-MOP-1′** and **Zr-MOP-10′** (Figures 1c, S9, S13, and S16). These results agree with the tetrahedral and dimeric assemblies being the kinetic and thermodynamic products of the reaction, respectively. Previous results have unveiled that the formation of each isomer type is dependent on the organic spacer length and bulkiness of substituents.^16^ In the case of **Zr-MOP-1-NH**_**2**_, only tetrahedral cages were isolated, probably due to the steric hindrance of the NH_2_ group.

The crystal and molecular structures of tetrameric **Zr-MOP-1-NH**_**2**_ and **Zr-MOP-10** and of dimeric **Zr-MOP-1′** have been univocally established using single-crystal X-ray diffraction (XRD) (Figures 1a–d and S9–S11). On the other hand, it was not possible to isolate good-quality single crystals of **Zr-MOP-1** and **Zr-MOP-10′**. The powder X-ray diffraction (PXRD) pattern of the former was compared to that of the NH_2_-functionalized analogue (Figure 1f). **Zr-MOP-1**, **Zr-MOP-1-NH**_**2**_, and **Zr-MOP-10** are isoreticular and crystallize in the cubic space group *Fm*3̅*m*. The unit cell [**Zr-MOP-1**: *a* = 37.131(2) Å and *V* = 51191(8) Å^3^, **Zr-MOP-1-NH**_**2**_: *a* = 36.777(3) Å and *V* = 49743(12) Å^3^, and **Zr-MOP-10**: *a* = 42.3762(5) Å and *V* = 76097(2) Å^3^] is composed by eight tetrahedral cages built around a central octahedral cavity (Figure 1a). Each cage is composed of four [(*n*-butylCpZr)_3_(OH)_3_O] Zr clusters connected by six dicarboxylate spacers, forming a tetrahedral-shaped cage with available internal volumes of ∼490 and ∼640 Å^3^ for **Zr-MOP-1**/**Zr-MOP-1-NH**_**2**_ and **Zr-MOP-10**, respectively. The triangular-shaped windows of the tetrahedra have apertures of ∼4.9 and ∼5.8 Å (for **Zr-MOP-1-NH**_**2**_ and **Zr-MOP-10**, respectively), enabling both the encapsulation of the 2-PAM drug (volume: 117.8 Å^3^)^17^ and the capture of the nerve agent simulant DIFP (volume: 148.9 Å^3^)^17^ (see below). The 12 externally dangling *n*-butyl groups generate a hydrophobic surface that ensures a higher solubility of these **Zr-MOPs** in organic solvents and/or biological tissues. On the other hand, the dimeric isomer **Zr-MOP-1′** crystallizes in the monoclinic space group *C*2/*c*. Each unit cell [*a* = 33.980(2) Å, *b* = 23.009(2) Å, *c* = 11.2581(8) Å, β = 97.560(3), and *V* = 8725.6(12) Å^3^] contains four cigar-like dimers with no accessible cavities (Figures 1c and S9). ^1^H NMR spectroscopy and ESI-MS are also diagnostic of the formation and stability of the tetrahedral cages. The results of ^1^H NMR in deuterated methanol and ESI-MS are indicative that the tetrahedral **Zr-MOP-1-X** (X = H and NH_2_) and **Zr-MOP-10** cages exist in the pure form (Figures 2, S12, S14, and S15), while in the case of the dimeric isomers **Zr-MOP-1′** and **Zr-MOP-10′**, only the latter is a pure system (Figures S13 and S16).

Regarding the thermal properties, **Zr-MOP-1**, **Zr-MOP-1-NH**_**2**_, and **Zr-MOP-10** are stable, under N_2_, up to approximately 185, 180, and 165 °C, respectively (Figures S26–S31). Moreover, in situ variable-temperature PXRD (VT-PXRD) (Figures S6–S8) demonstrated that solvent loss is not accompanied by the loss of crystallinity or phase transition. These results confirm that the integrity of the tetrahedral cages is maintained after thermal activation, which is in line with the behavior of other robust noncovalent porous materials.^18^

### Host–Guest Chemistry

N_2_ adsorption at 77 K was used to explore the accessibility of the empty volume of the tetrahedral cages. The results are in agreement with crystalline microporous systems exhibiting type I isotherms with Brunauer–Emmett–Teller (BET) values of 435 and 1140 m^2^ g^–1^ for **Zr-MOP-1** and **Zr-MOP-10**, respectively (Figure 1g). These values are about one-half of those found for the related extended MOF materials **UiO-66** and **UiO-67**, respectively, which agrees with the loss of the octahedral pore contribution to the porosity of these materials.^19^ Additionally, the very low porosity observed for **Zr-MOP-1-NH**_**2**_ can be attributed to the incorporation of the amino residues in the organic linker benzene-2-amino-1,4-dicarboxylate, which leads to the blocking of N_2_ diffusion into the tetrahedral cages. In this case, CO_2_ at 195 K, with a higher kinetics energy of adsorption, is able to diffuse into the cages, giving rise to a moderate BET surface area of 177 m^2^ g^–1^ (Figure S32).

**Figure 3:**
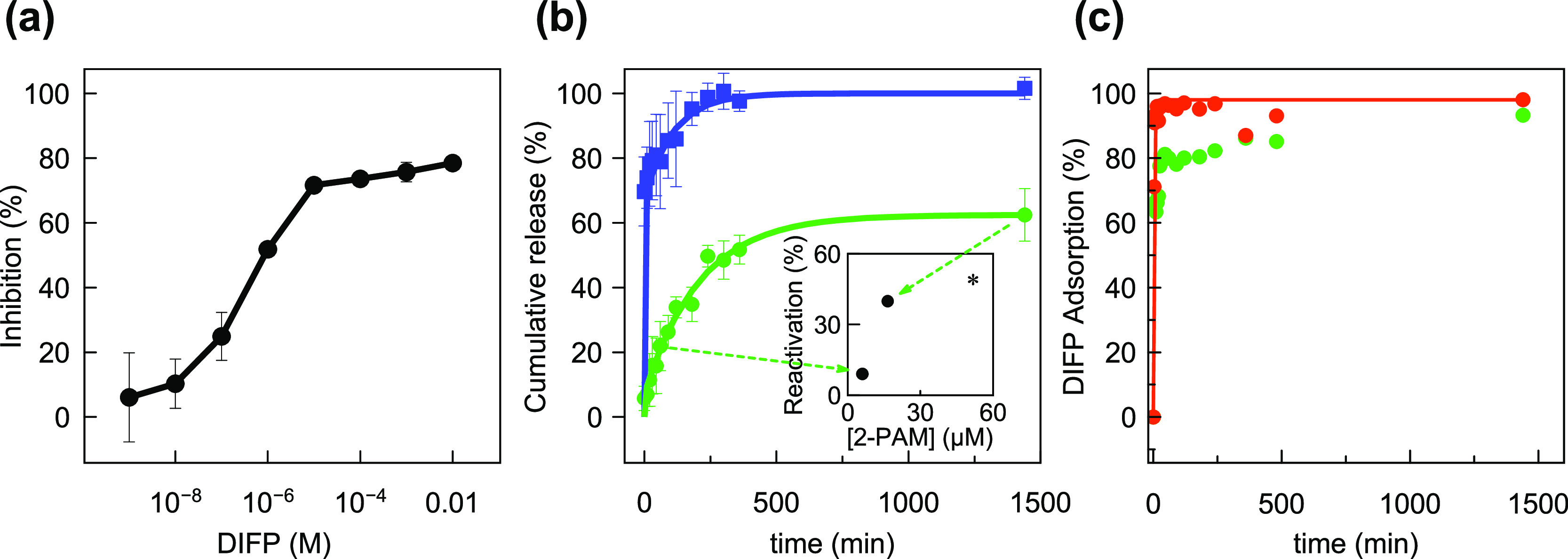
(a) AChE inhibition profile by DIFP in tris–HCl buffer (pH = 7.4); (b) 2-PAM release from **2-PAM@Zr-MOP-1** (green circles) and **2-PAM@Zr-MOP-10** (blue squares); inset in graph b corresponds to AChE reactivation by 2-PAM supernatants released by **2-PAM@Zr-MOP-1**, with the asterisk standing for AChE reactivation by 50 μM free 2-PAM; and (c) DIFP (14.4 mM) capture by **Zr-MOP-1** (red) and **2-PAM@Zr-MOP-1** (green). Experimental conditions.

Once we proved the stability and permanent porosity of the **Zr-MOP-1** and **Zr-MOP-10** cages, we explored their suitability as dual materials for nerve agent intoxication treatment (Scheme 1). In the first step, we found that these systems are suited for the fast and efficient capture of model organophosphate DIFP molecules from aqueous solutions (14.4 mM) with respective half-life times *t*_1/2_ of 4.2 and 16.5 min for **Zr-MOP-1** and **Zr-MOP-10** (Figures 3c and S37). DIFP incorporation into the cages is confirmed using ^31^P NMR and computational modeling, with no hydrolytic degradation being observed (Figures S38–S40). This behavior differs from that found on the structure of the related extended **UiO-6X** systems, which are able to hydrolyze P–X (X = F, O, and S) bonds of nerve agents/pesticides.^20^ The lack of hydrolytic activity can be attributed to the lower Lewis acidity and/or steric hindrance of the *n*-butylCpZr residues in these **Zr-MOPs**.

In the second step, we explored the suitability of the **Zr-MOPs** for the encapsulation of the AChE reactivator pralidoxime drug (2-PAM). With this aim, freshly prepared **Zr-MOP** crystals were soaked into a 2-PAM 70 mM DMF solution (1:7 M ratio) and left to equilibrate for 1 week at room temperature. After this period, the crystals were recovered and thoroughly washed (Figures S4 and S5). ^1^H NMR results in deuterated methanol are indicative of the formation of 1:1 **2-PAM**@**Zr-MOP-1(0)** assemblies with the 2-PAM signals being shifted upfield (0.02 ppm), suggesting its incorporation into the MOP cages (Figures 2b and S17–S20). It is noteworthy that ESI-MS spectra show a peak at 3934.36 *m*/*z*, corresponding to the **Zr-MOP-1-4Cl-6H+2-PAM**^**+**^ assembly (Figures 2c, S25), which is diagnostic of drug incorporation. The affinity of 2-PAM for **Zr-MOP-1(0)** is further supported by computational modeling, which shows the accommodation of one drug molecule into the cage of **Zr-MOP-1(0)** systems (Figures 2a and S41).

### Biophysical Studies

As mentioned above, a sustained concentration of 2-PAM is necessary in order to achieve a proper organophosphate intoxication treatment. With this aim, we have studied 2-PAM controlled release from **2-PAM**@**Zr-MOP-1(0)** assemblies under simulated biological conditions, using Tris–HCl buffered aqueous suspensions (pH 7.4). The release profiles for **2-PAM**@**Zr-MOP-1** and **2-PAM**@**Zr-MOP-10** systems can be adjusted to a pseudo-first-order kinetics model (Figure 3b). The results show a gradual release of 2-PAM, with a *t*_1/2_ of 312 min for **2-PAM**@**Zr-MOP-1**, reaching about 60% cumulative liberation after 24 h. In the case of **2-PAM**@**Zr-MOP-10**, a burst release of the drug (approx. 70% of encapsulated 2-PAM) is followed by a slower release, reaching 80% after 53 min and 100% delivery after 7 h. It is noteworthy that 2-PAM incorporation into **Zr-MOP-1** seems to have a moderate impact on the fast and efficient DIFP capture, with t_1/2_ increasing from 4.2 to 15.8 min on passing from **Zr-MOP-1** to the **2-PAM**@**Zr-MOP-1** assembly (Figure 3c). Taking into account the dual behavior of **2-PAM@Zr-MOP-1** for DIFP capture and controlled drug release, we have selected this platform for AChE reactivation assays (see below). The ultimate goal of this study was to explore the suitability of the **2-PAM@Zr-MOP-1** assemblies in the reactivation of DIFP-inhibited AChE as a proof of concept of their suitability for organophosphate intoxication treatment. With this aim, we first evaluated the AChE activity using indoxyl acetate as the enzyme substrate in Tris–HCl buffered (pH 7.4) aqueous media. DIFP addition leads to AChE inhibition, with 50% of activity being reached for a 5 × 10^–6^ M concentration of the nerve agent simulant (Figure 3a). This study was followed by the evaluation of the AChE reactivation ability of the **2-PAM@Zr-MOP-1** assembly. In a typical experiment, 50% inhibited AChE was exposed to 2-PAM supernatants released from **2-PAM@Zr-MOP-1** suspensions after 1 h (6.06 × 10^–6^ M) and 24 h (1.65 × 10^–5^ M) of incubation in Tris–HCl buffer. The results show a 9 ± 1% and 40 ± 7% of AChE reactivation, respectively (inset in Figure 3b). It is noteworthy that a control reactivation assay using 5 × 10^–5^ M of 2-PAM alone, in the same concentration range as that of 2-PAM released by **2-PAM@Zr-MOP-1** after 24 h, gives rise to a 48 ± 6% reactivation. This suggests that **Zr-MOP-1** does not negatively affect the ability of the released oxyme to reactivate AChE. As mentioned before, the **2-PAM@Zr-MOP-1** assembly is still able to efficiently capture DIFP (Figure 3c), endowing this system with dual properties for organophosphate poisoning treatment before and after the toxic molecule reaches its biological target.

**Figure 4:**
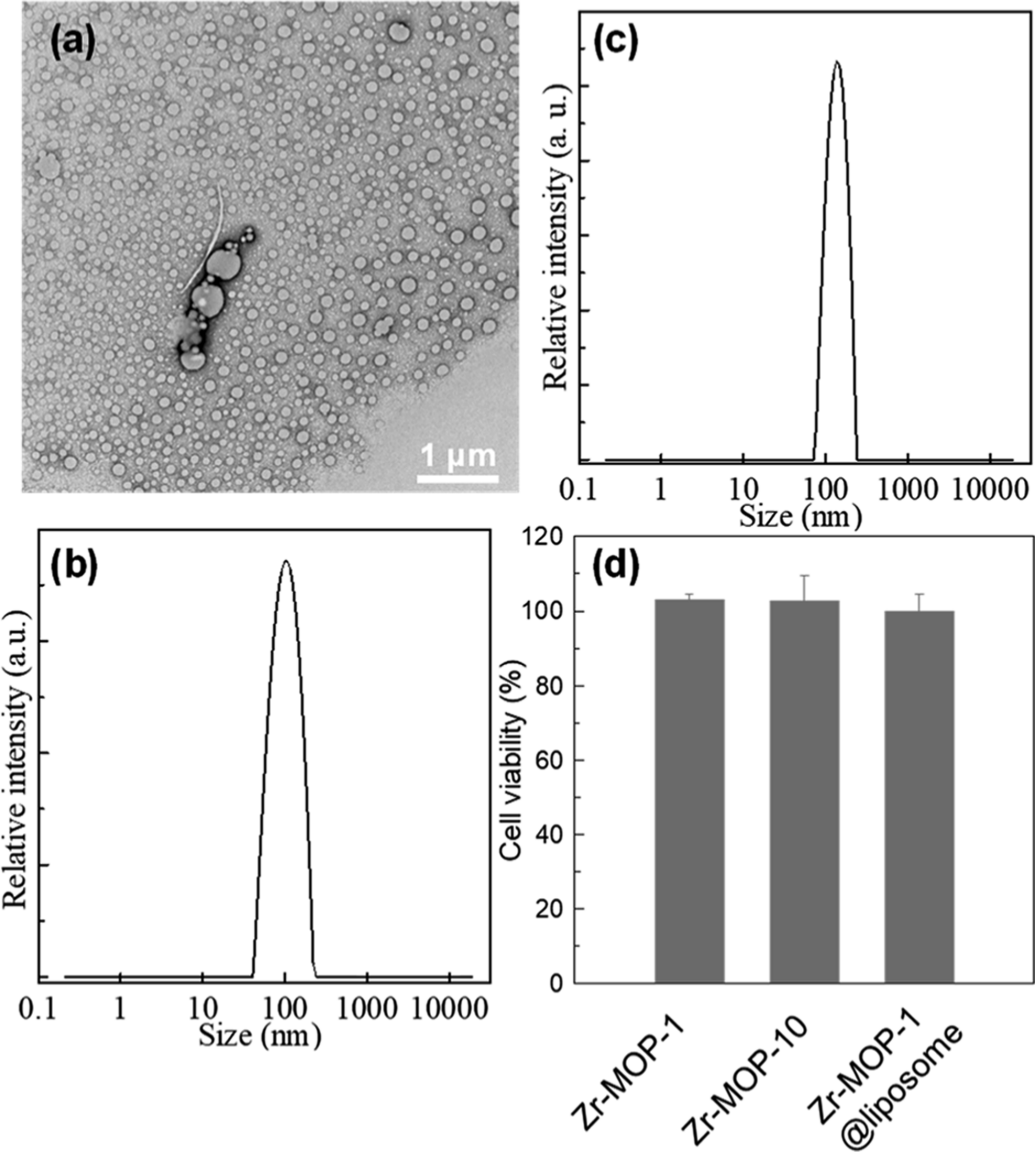
TEM images (a) and DLS size distribution (b) of **2-PAM@Zr-MOP-1@liposome** prepared from a **2-PAM@Zr-MOP-1** solution in CHCl_3_. (c) DLS size distribution of **2-PAM@Zr-MOP-1@liposome** prepared from a **2-PAM@Zr-MOP-1** solution in MeOH. (d) Cell viability of SH-SY5Y human neuroblastoma after 24 h incubation with **Zr-MOP-1** (10 μM), **Zr-MOP-10** (10 μM), and **2-PAM@Zr-MOP-1@liposome** (5 μM for **Zr-MOP-1**).

The processing of both chloroform and methanol solutions of **2-PAM**@**Zr-MOP-1** with the phosphatidylcholine surfactant leads to aqueous colloidal dispersions of biocompatible liposomes. Transmission electron microscopy (TEM) (Figures 4a and S42) and dynamic light scattering (DLS) (Figure 4b,c) studies confirm the formation of **2-PAM**@**Zr-MOP-1**@**liposome** assemblies of 200 nm (processing from chloroform) and 100 nm (processing from methanol) mean sizes. In addition, TEM–EDX analyses suggest that **Zr-MOP-1** cages are mainly associated to the liposome lipid layer (Figures S43 and 44). It is noteworthy that the hybrid **2-PAM**@**Zr-MOP-1**@**liposome** ([2-PAM] = 5 × 10^–6^ M) assemblies are able to give rise to a 10% reactivation of DIFP-inhibited AChE. This result paves the way for the use of **Zr-MOPs** as drug delivery platforms for organophosphate treatment. Finally, we have carried out in vitro neurotoxicity tests toward SH-SY5Y human neuroblastoma cell culture.^21^ The results indicate that the neuroblastoma cell viability is unaffected by **2-PAM**@**Zr-MOP-1** assemblies before and after being incorporated into phosphatidylcholine liposomes (Figure 4d). These experiments show that the assayed platforms for AChE reactivation are not neurotoxic and might become suitable for reversing the nerve system damage of organophosphate poisoning.

## Conclusions

As a takeaway message, we have shown that the decoration of the Cp-capping ligands with alkyl residues in **Zr-MOPs** leads to soluble and biocompatible materials. It is noteworthy that the reported systems are able to capture the nerve agent simulant DIFP and incorporate and release, in a controlled manner, 2-PAM, leading to the concomitant reactivation of DIFP-inhibited AChE. In addition, these systems can be incorporated into non-neurotoxic biocompatible liposome assemblies, opening the way to their use as drug vehicles for organophosphate poisoning treatment. Altogether, these results can be considered as a proof of concept of the possible utility of **Zr-MOPs** as dual systems for poisoning treatments (beyond nerve agents/pesticides), being able to both capture a toxin and reactivate its biological target.

## Supporting Information Available

Synthetic protocols, full characterization of Zr-MOP materials, details of PAM incorporation and release and AChE inhibition, reactivation assays, liposome preparation, and cell viability studies. CCDC 2153759, 2153760 and 2153911 contains the supplementary crystallographic data for this paper. These data can be obtained free of charge from The Cambridge Crystallographic Data Centre via www.ccdc.cam.ac.uk/ (PDF)
