# Supplementary material for: Zirconium Metal–Organic Polyhedra with Dual Behavior for Organophosphate Poisoning Treatment
Source: ACS Appl Mater Interfaces. 2022 Jun 2;14(23):26501–6. doi: 10.1021/acsami.2c06025 (PMC9204697; doi:10.1021/acsami.2c06025)
Supplement: Supplementary file 1 — am2c06025_si_001.pdf [file am2c06025_si_001.pdf]

SUPPORTING INFORMATION

Zirconium metal-organic polyhedra with dual behavior for organophosphate poisoning treatment

Pedro Delgado[#,1], Javier D. Martin-Romera[#,1], Cristina Perona[#], Rebecca Vismara[#,±], Simona Galli[±], Carmen R. Maldonado[#], Francisco J. Carmona[#]*, Natalia M. Padial[ϕ]* and Jorge A. R. Navarro[#]*

[#]Departamento de Química Inorgánica, Universidad de Granada, Av. Fuentenueva S/N, 18071, Granada, Spain.

[±]Dipartimento di Scienza e Alta Tecnologia, Università dell'Insubria, Via Valleggio 11, 22100 Como, Italy

[ϕ]Functional Inorganic Materials Team, Instituto de Ciencia Molecular (ICMol), Universitat de València, 46980 València, Spain

[1]Equal contribution

Correspondence to:** *jarn@ugr.es*; *fjcarmona@ugr.es*; *natalia.munoz@uv.es

Index

S.1. General Considerations......3

S.2. Synthesis of compounds. 4

S.3. Physical and chemical characterization. 9

S.4. DIFP adsorption studies......42

S.5. UV-Vis assays......46

S.6. Computational modelling (Adsorbate locator)......47

S.7. Transmission electron microscopy (TEM)......48

S.1. General Considerations.

S.1.1. Materials and reagents.

All chemicals and solvents were commercially available at commercial sources and used without further purification.

S.1.2. Physical and chemical methods of characterization.

Powder X-ray diffraction data (PXRD) were obtained at room temperature on a Bruker D2 PHASER diffractometer using Cu Kα radiation (λ = 1.5418 Å), collecting in the 3-25° 2θ range, with steps of 0.02° and time per step of 0.5 s. The samples were deposited in the hollow of a zero-background silicon sample holder. The thermal behaviour of the studied Zr-MOPs was investigated by in situ variable-temperature powder X-ray diffraction (VT-PXRD). VT-PXRD analyses were carried out with a Bruker AXS D8 Advance diffractometer (λ = 1.5418 Å). 20 mg of the as-synthesized compounds were heated in air from 25 °C until decomposition (T_{dec} = 170-200 °C) with steps of 10 °C using a custom-made sample heater (Officina Elettrotecnica di Tenno, Ponte Arche, Italy). A PXRD pattern was acquired at each step, covering a sensible low-to-medium-angle 2θ range, with steps of 0.02°, and time per step of 1 s. Whole powder pattern parametric refinements of the data acquired before the loss of crystallinity disclosed the behaviour of the unit cell parameters as a function of temperature. ^{1}H and ^{31}P Nuclear Magnetic Resonance Spectroscopy (^{1}H- and ^{31}P-NMR) data were obtained with a BRUKER Nanobay Avance III HD High Definition 400 MHz (2-channel) NMR spectrometer. Thermogravimetric analysis (TGA) was carried out by a thermogravimetric analyser METTLER-TOLEDO mod. TGA/DSC1. Nitrogen adsorption isotherms at 77 K and CO_2 adsorption isotherms at 195 K were measured on a Micromeritics 3Flex adsorption analyzer. Fourier Transform Infrared Spectroscopy (FTIR) measurements were obtained with a Bruker spectrophotometer with an ATR module. Scanning Electron Microscopy–Energy Dispersive X-Ray (SEM-EDX) images and element mapping were obtained on a Hitachi S-4800 instrument. Transmission Electron Microscopy (TEM) images were acquired on a LIBRA 120 PLUS Carl Zeiss SMT microscope. DIFP adsorption experiments were carried out in an Agilent 8860 GC chromatograph. This chromatograph has a FID detector and a 16 port autosampler. A HP-5 column of 50 m length, 0.320 mm diameter and 1.05 μm thickness was used, which allowed working from -60 ºC to 325 ºC. Enzymatic assays were performed in a Tecan Infinite® 200 PRO NanoQuant.

S.2. Synthesis of compounds.

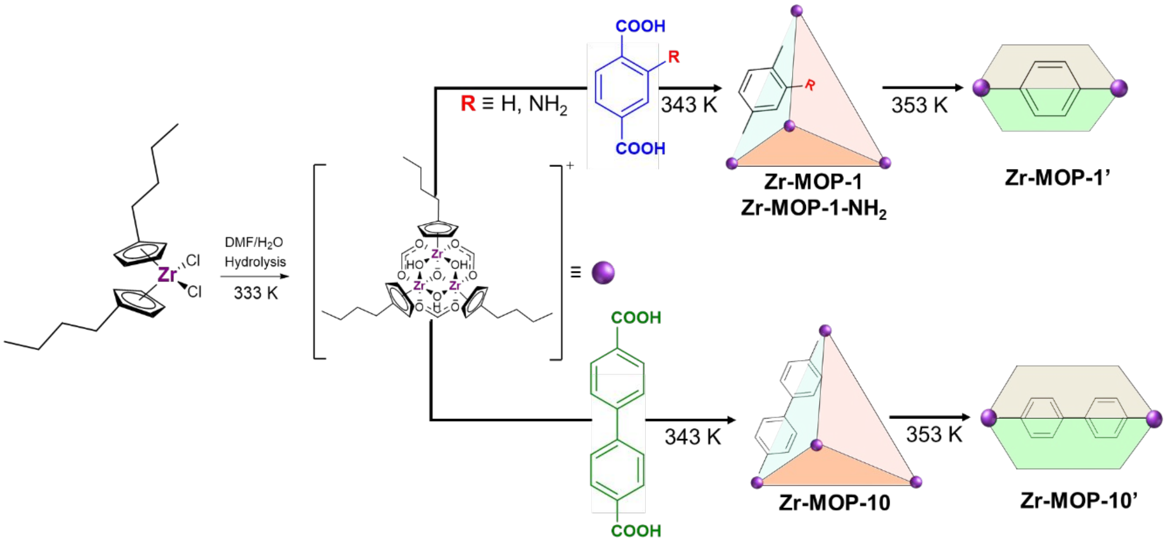

Scheme S1. Schematic representation of the synthesis of *n*-butyl functionalized tetrahedral metal-organic polyhedra **Zr-MOP-1(0)-X** and their dimeric **Zr-MOP-1(0)'** isomers. X = H, NH_2.

S.2.1. Zr-MOP-1 $[(Zr_3(\mu_2\text{-}OH)_3(\mu_3\text{-}O)(n\text{-}butylCp)_3)_x(C_8H_4O_4)_n]Cl_x$ crystals. x = 6, n =6 for the cubic phase and x = 2, n = 3 for the monoclinic phase.

In a 4 mL teflon reactor, 4.43 mg of terephthalic acid (0.027 mmol) and 21.4 mg of 1,1´-dibutylzirconocene dichloride ($[Zr(n\text{-}butylCp)_2Cl_2]$ (0.053 mmol)) were dissolved in 2 mL of N,N-dimethylformamide (DMF) and 0.5 mL of water. The mixture was heated at 70 °C during 8 h (heating-ramp of 2 °C/min, cooling ramp of 0.1 ºC/min). Cubic and prismatic crystals of two different phases, **Zr-MOP-1** and **Zr-MOP-1'** respectively, were collected and analysed by single-crystal X-ray diffraction.

S.2.2. Zr-MOP-1 $[(Zr_3(\mu_2\text{-}OH)_3(\mu_3\text{-}O)(n\text{-}butylCp)_3)_4(C_8H_4O_4)_6]Cl_6$ (bulk).

In a 10 mL glass vial, 27 mg of terephthalic acid (0.169 mmol) and 132 mg of 1,1´-dibutylzirconocene dichloride (0.326 mmol) were dissolved in 2 mL of N,N-dimethylformamide and 1.5 mL of water. The solution was heated at 65 °C during 8 h. The resulting solid was washed with DMF (3 × 3.5 mL) and *n*-hexane (1 × 3.5 mL) and recovered by centrifugation (4000 rpm, 10 min). Finally, the solid was soaked in *n*-hexane for 24 hours and activated under dynamic vacuum (60 ºC, 8 h). Yield: 90.6 mg, 83.3%. ^1H NMR (400.1 MHz, CD_3OD): δ (ppm) 8.07 (s, 24 H, Ph), 8.00 (s, CHO, DMF), 6.56

(t, J = 4 Hz, 24 H, Cp), 6.40 (t, J = 4 Hz, 24 H, Cp), 3.01 (s, CH_3, DMF), 2.88 (s, CH_3, DMF), 2.84 (t, J = 8 Hz, 26 H, CH_2, *n*-butyl), 1.71 (q, J = 8 Hz, 26 H, CH_2, *n*-butyl), 1.46-1.37 (m, 25 H, CH_2, *n*-butyl), 0.98 (t, J = 8 Hz, 39 H, CH_3, *n*-butyl).

S.2.3. Zr-MOP-1' $[(Zr_3(\mu_2\text{-}OH)_3(\mu_3\text{-}O)(n\text{-}butylCp)_3)_x(C_8H_4O_4)_n]Cl_x$ (bulk) x = 6, n = 6 for the cubic phase and x = 2, n = 3 for the monoclinic phase.

In a 5 mL glass vial, 4.43 mg of biphenyl-4,4′-dicarboxylic acid (0.0267 mmol) and 21.4 mg of 1,1′-dibutylzirconocene dichloride (0.053 mmol) were dissolved in 2 mL of DMF and 0.25 mL of water. The solution was heated at 80 °C during 8 h. The resulting solid was washed with DMF (3 × 3.5 mL) and acetone (1 × 3.5 mL), recovered by centrifugation (4000 rpm, 10 min) and dried under air. Yield: 9.1 mg, 33.3% (monoclinic phase), 19.5% (tetrahedral phase), determined by NMR. ^1H NMR (400.1 MHz, CD_3OD): δ (ppm) 8.07 (s, 9 H, Ph, cubic phase), 8.00 (s, CHO, DMF), 7.74 (s, 15 H, Ph, monoclinic phase), 6.56 (t, J = 4 Hz, 24 H, Cp), 6.40 (t, J = 4 Hz, 24 H, Cp), 3.01 (s, CH_3, DMF), 2.88 (s, CH_3, DMF), 2.84 (t, J = 8 Hz, 26 H, CH_2, *n*-butyl), 1.71 (q, J = 8 Hz, 26 H, CH_2, *n*-butyl), 1.48-1.38 (m, 27 H, CH_2, *n*-butyl), 0.98 (t, J = 8 Hz, 38 H, CH_3, *n*-butyl).

S.2.4. Zr-MOP-1-NH_2 $[(Zr_3(\mu_3\text{-}OH)_3(\mu_2\text{-}O)(n\text{-}butylCp)_3)_4(C_8H_5O_4N)_6]Cl_6$ crystals.

In a 4 mL teflon reactor, 4.91 mg of 2-aminoterephthalic acid (0.027 mmol) and 21.4 mg of 1,1′-dibutylzirconocene dichloride (0.053 mmol) were dissolved in 2 mL of DMF and 0.25 mL of water. The mixture was heated at 70 °C during 8 h (heating-ramp of 2 °C/min, cooling ramp of 0.1 ºC/min). Cubic crystals, corresponding to the cubic crystalline phase, were collected and analysed by single-crystal X-ray diffraction. No appearance of the monoclinic phase was observed.

S.2.5. Zr-MOP-1-NH_2 $[(Zr_3(\mu_3\text{-}OH)_3(\mu_2\text{-}O)(n\text{-}butylCp)_3)_4(C_8H_5O_4N)_6]Cl_6$ (bulk).

In a 10 mL glass vial, 57.7 mg of 2-aminoterephthalic acid (0.318 mmol) and 250 mg of 1,1′-dibutylzirconocene dichloride (0.618 mmol) were dissolved in 5.7 mL of DMF and 2.85 mL of water. The solution was heated for 8 h at 70 °C. The resulting solid was washed with DMF (3 × 10 mL) and acetone (1 × 10 mL) and recovered by centrifugation (4000 rpm, 10 min). The washed solid was soaked in *n*-hexane during 24 h. To remove

the solvent trapped inside the pores, the material was activated under dynamic vacuum at 60 ºC during 8 h. Yield: 202.4 mg, 96.4%. ^{1}H NMR (400.1 MHz, CD_3OD): δ (ppm): 8.00 (s, CHO, DMF), 7.84-7.82 (m, Ph), 7.35-7.32 (m, Ph), 7.16 (s broad, Ph), 6.54-6.50 (m, Cp), 6.42 (s broad, Cp), 3.01 (s, CH_3, DMF), 2.88 (s, CH_3, DMF), 2.75-2.65 (m, CH_2, *n*-butyl), 1.63 (m, CH_2, *n*-butyl), 1.35 (CH_2, *n*-butyl), 0.93 (m, CH_3, *n*-butyl).

S.2.6. Zr-MOP-10 $[(Zr_3(\mu_2\text{-}OH)_3(\mu_3\text{-}O)(n\text{-butylCp})_3)_x(C_{14}H_8O_4)_n]Cl_x$ crystals. x = 6, n = 6 for the cubic phase and x = 2, n = 3 for the monoclinic phase.

In a 4 mL teflon reactor, 4.43 mg of biphenyl-4,4′-dicarboxylic acid (0.041 mmol) and 21.4 mg of 1,1´-dibutylzirconocene dichloride (0.053 mmol) were dissolved in 2 mL of DMF and 0.25 mL of water. The mixture was heated at 70 °C during 12 h (heating-ramp of 2 °C/min, cooling ramp of 0.1 ºC/min). Cubic and prismatic crystals of two different phases, **Zr-MOP-10** and **Zr-MOP-10'** respectively, were collected and analysed by single-crystal X-ray diffraction.

S.2.7. Zr-MOP-10 $[(Zr_3(\mu_2\text{-}OH)_3(\mu_3\text{-}O)(n\text{-butylCp})_3)_4(C_{14}H_8O_4)_6]Cl_6$ (bulk).

In a 5 mL glass vial, 20 mg of biphenyl-4,4′-dicarboxylic acid (0.185 mmol) and 55.5 mg of 1,1´-dibutylzirconocene dichloride (0.137 mmol) were dissolved in 0.45 mL of N,N-dimethylformamide, 0.22 mL of carbon tetrachloride and 0.4 mL of water. The mixture was heated at 60 °C for 12 h. The resulting suspension was washed with DMF (3 × 3 mL) and *n*-hexane (1 × 3 mL) and recovered by centrifugation (4000 rpm, 10 min). The washed solid was soaked in *n*-hexane during 24 h. To remove the solvent trapped inside the pores, the material was activated under dynamic vacuum at 60 ºC during 8 h. Yield: 45.7 mg, 92.3%. ^{1}H NMR (400.1 MHz, CD_3OD): δ (ppm): 8.04 (d, J = 8 Hz, 24 H, Ph), 8.00 (s, CHO, DMF), 7.81 (d, J = 8 Hz, 24 H, Ph), 6.56 (t, J = 4 Hz 24 H, Cp), 6.40 (t, J = 4 Hz 24 H, Cp), 3.01 (s, CH_3, DMF), 2.87 (s, CH_3, DMF), 2.85 (t, J = 8 Hz, 27 H, CH_2, *n*-butyl), 1.70 (q, J = 8 Hz, 30 H, CH_2, *n*-butyl), 1.45-1.36 (m, 31 H, CH_2, *n*-butyl), 0.96 (t, J = 8 Hz, 42 H, CH_3, *n*-butyl).

S.2.8. Zr-MOP-10' $[(Zr_3(\mu_2\text{-}OH)_3(\mu_3\text{-}O)(n\text{-butylCp})_3)_2(C_{14}H_8O_4)_3]Cl_2$ (bulk).

In a 5 mL glass vial, 20 mg of biphenyl-4,4′-dicarboxylic acid (0.185 mmol) and 55.5 mg of 1,1′-dibutylzirconocene dichloride (0.137 mmol) were dissolved in 0.45 mL DMF, 2 mL of carbon tetrachloride and 0.5 mL of water. The solution was heated at 80 °C for 12 h. Then, the powder were obtained by vapor diffusion with diethyl ether. They were washed with DMF (3 × 3 mL) and acetone (1 v 3 mL) and dried under air. Yield: 34.1 mg, 67.7%. ^{1}H NMR (400.1 MHz, CD_3OD): δ (ppm): 8.00 (s, CHO, DMF), 7.70 (d, J = 8 Hz, 24 H, Ph), 7.39 (d, J = 8 Hz, 24 H, Ph), 6.56 (t, J = 4 Hz 24 H, Cp), 6.40 (t, J = 4 Hz, 24 H, Cp), 3.01 (s, CH_3, DMF), 2.88-2.86 (m, CH_3, DMF and CH_2, *n*-butyl), 1.71 (q, J = 8 Hz, 26 H, CH_2, *n*-butyl), 1.48-1.39 (m, 27 H, CH_2, *n*-butyl), 0.98 (t, J = 8 Hz, 37 H, CH_3, *n*-butyl).

S.2.9. PAM encapsulation into Zr-MOP-1 (Zr-MOP-1@2-PAM).

Fresh **Zr-MOP-1** was mixed with 23.39 mg of pralidoxime (0.136 mmol) and 5.44 mg of sodium hydroxide (0.136 mmol) in 2.5 mL of DMF. After one week at room temperature, the solid was washed with water (2 × 3.5 mL) and acetone (1 × 3.5 mL) and recovered by centrifugation. The resulting material was heated at 60 °C for 2 hours. ^{1}H NMR (400.1 MHz, CD_3OD): δ (ppm) 8.86 (d, py, pralidoxime), 8.69 (s, CH_2=NOH, pralidoxime), 8.54-8.49 (m, py, pralidoxime), 8.07 (s, Ph, **Zr-MOP-1**), 8.00 (s, CHO, DMF), 6.57 (t, Cp), 6.40 (t, Cp), 4.44 (s, Me, pralidoxime), 3.01 (s, CH_3, DMF) 2.84 (t, CH_2, *n*-butyl, **Zr-MOP-1**), 2.72 (s, CH_3, DMF) 1.71 (q, CH_2, *n*-butyl, **Zr-MOP-1**), 1.46-1.37 (m, CH_2, *n*-butyl, **Zr-MOP-1**), 0.98 (t, CH_3, *n*-butyl, **Zr-MOP-1**).

S.2.10. PAM encapsulation into Zr-MOP-10 (Zr-MOP-10@2-PAM).

The procedure to encapsulate 2-PAM inside the cavities of **Zr-MOP-10** was similar to that performed for **Zr-MOP-1**, except that only 9.85 mg of pralidoxime (0.057 mmol) were mixed with fresh **Zr-MOP-10** in 2.5 mL of DMF. ^{1}H NMR (400.1 MHz, CD_3OD): δ (ppm) 8.86 (d, py, pralidoxime), 8.69 (s, CH_2=NOH, pralidoxime), 8.54-8.49 (m, py, pralidoxime), 8.04 (d, Ph, **Zr-MOP-10**), 8.00 (s, CHO, DMF), 7.81 (d, Ph, **Zr-MOP-10**), 6.56 (t, Cp, **Zr-MOP-10**), 6.40 (t, Cp, **Zr-MOP-10**), 4.43 (s, CH_3, pralidoxime), 3.01 (s, CH_3, DMF), 2.88 (s, CH_3, DMF), 2.84 (t, CH_2, *n*-butyl, **Zr-MOP-10**), 1.70 (q, CH_2, *n*-

butyl, **Zr-MOP-10**), 1.43-1.36 (m, CH_2, *n*-butyl, **Zr-MOP-10**), 0.96 (t, CH_3, *n*-butyl, **Zr-MOP-10**).

S.2.11. Preparation of liposome

Giant unilamellar vesicles (GUV) were prepared as previously reported.[1] 10 mg of egg yolk phosphatidylcholine (EYPC) and 1 mg of **Zr-MOP-1@2-PAM** were dissolved in 10 mL of chloroform. The solvent was removed under reduced pressure during 15 min and the resulting film was dried overnight, under dynamic vacuum. Afterwards, the film was hydrated at 37 ºC during 12 h with 5 mL of sucrose aqueous solution (0.2 M). The sample was treated with uranyl acetate for TEM images acquisition.

S.3. Physical and chemical characterization.

S.3.1. Powder X-Ray Diffraction (PXRD).

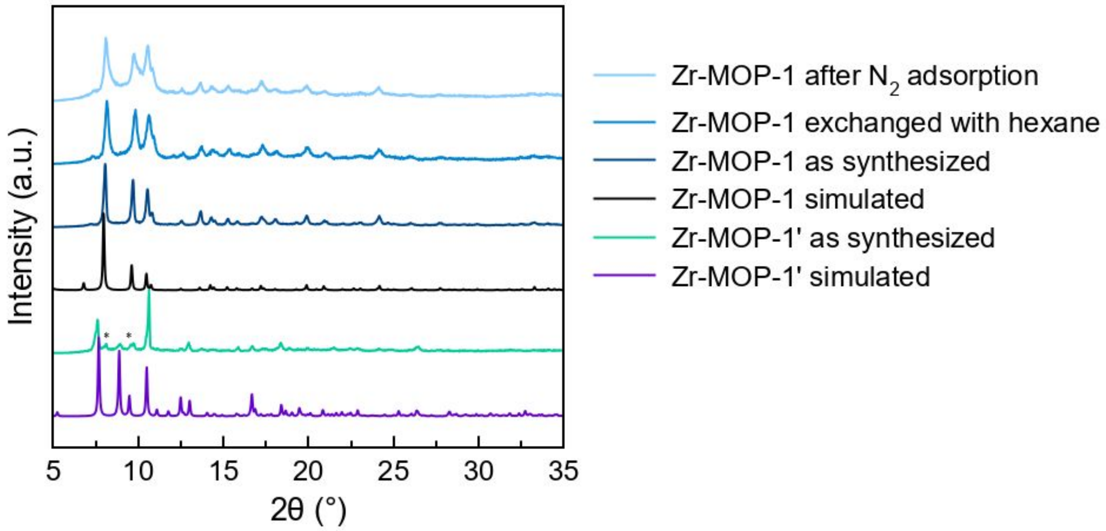

Figure S1. Comparison of the experimental PXRD patterns of **Zr-MOP-1'** as synthesized and **Zr-MOP-1** as synthesized, exchanged with hexane and after N_2 adsorption, with the simulated ones of **Zr-MOP-1** and **Zr-MOP-1'**. The stars indicate an impurity in the batch of the cubic phase.

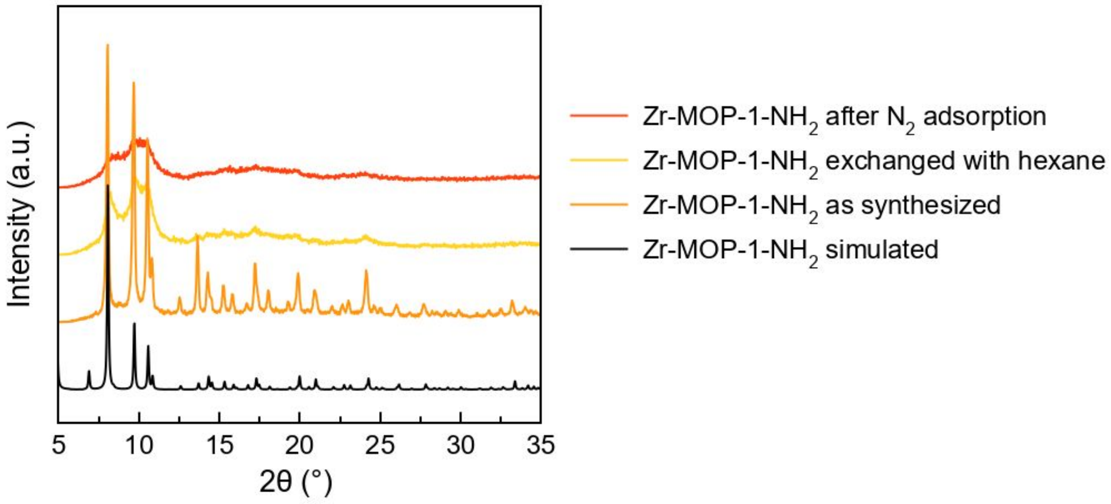

Figure S2. Comparison of the experimental PXRD patterns of **Zr-MOP-1-NH$_2$** as synthesized, exchanged with hexane and after N_2 adsorption with the simulated one of **Zr-MOP-1-NH$_2$**.

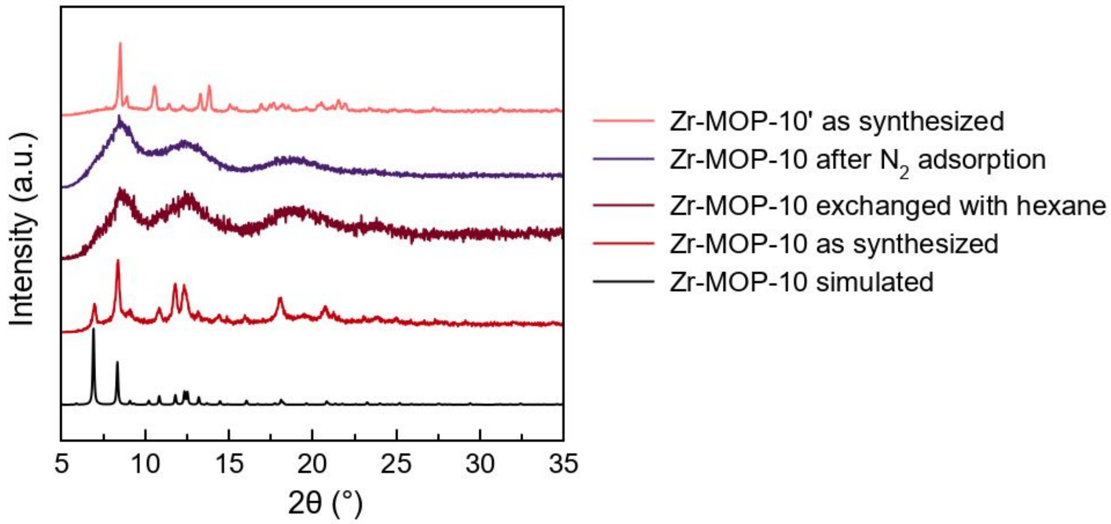

Figure S3. Comparison of the experimental PXRD patterns of **Zr-MOP-10'** as synthesized and **Zr-MOP-10** as synthesized, exchanged with hexane and after N_2 adsorption with the simulated one of **Zr-MOP-10**.

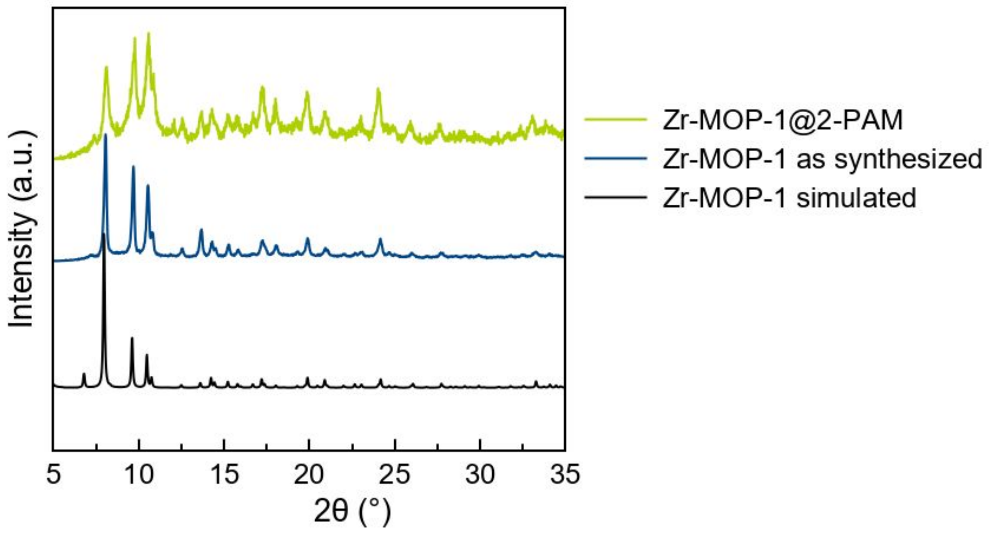

Figure S4. Comparison of the experimental PXRD patterns of **Zr-MOP-1@2-PAM** (top) and **Zr-MOP-1** (middle) with the simulated one of **Zr-MOP-1** (bottom).

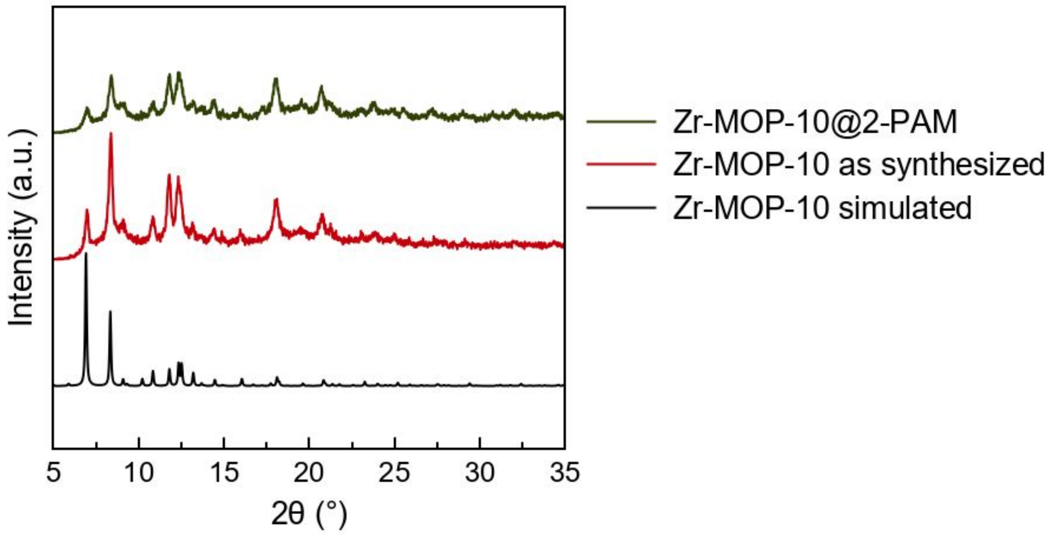

Figure S5. Comparison of the experimental PXRD patterns of **Zr-MOP-10@2-PAM** (top) and **Zr-MOP-10** (middle) with the simulated one of **Zr-MOP-10** (bottom).

S.3.2. Variable-Temperature Powder X-Ray Diffraction (VT-PXRD).

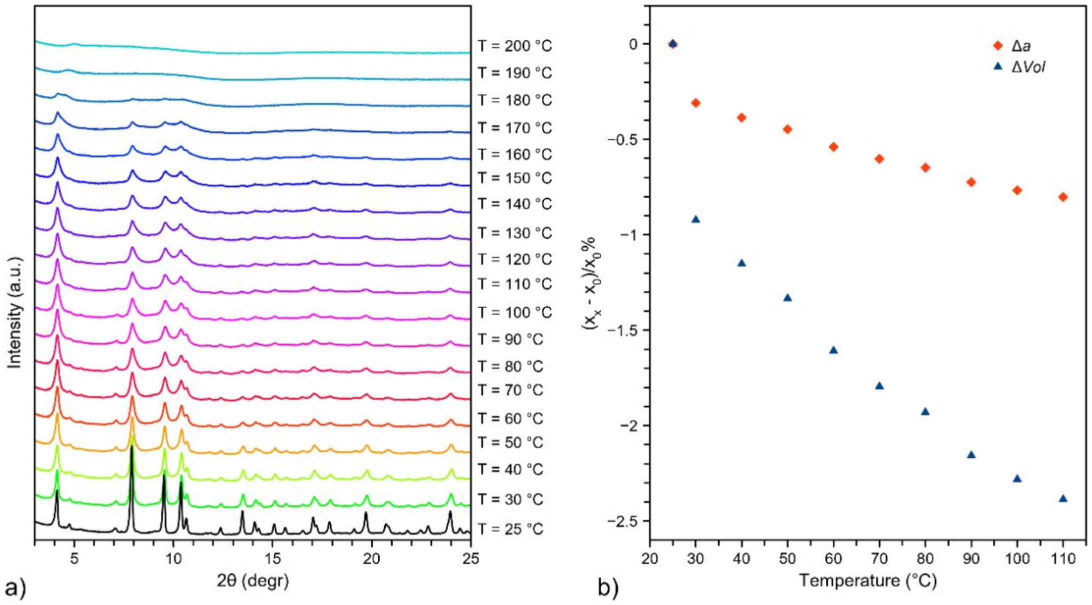

Figure S6. a) Powder X-ray diffraction patterns (Cu Kα, λ = 1.5418 Å) measured on as-synthesized **Zr-MOP-1** as a function of temperature heating in air, with steps of 10 °C, in the temperature range 25–200 °C. (b) Percentage variation of the unit cell parameters of **Zr-MOP-1** during the VT-PXRD experiment with respect to the T = 25 °C ones, as result of the parametric whole powder pattern refinement carried out with the Le Bail approach.

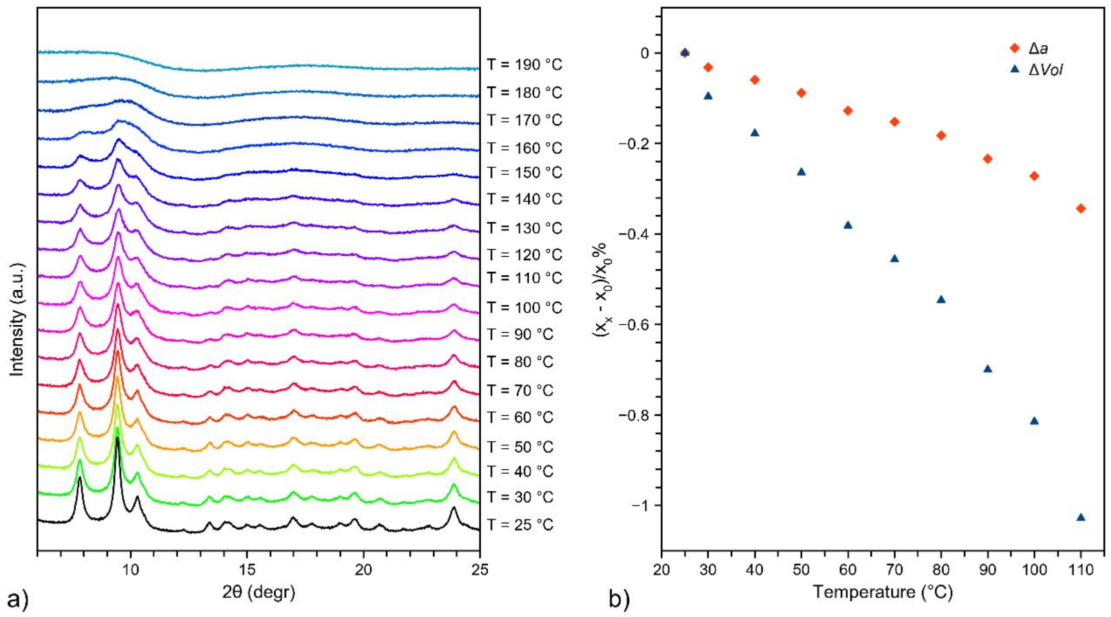

Figure S7. a) Powder X-ray diffraction patterns (Cu Kα, λ = 1.5418 Å) measured on as-synthesized **Zr-MOP-1-NH$_2$** as a function of temperature heating in air, with steps of 10 °C, in the temperature range 25–190 °C. (b) Percentage variation of the unit cell parameters of **Zr-MOP-1-NH$_2$** during the VT-PXRD experiment with respect to the T = 25 °C ones, as result of the parametric whole powder pattern refinement carried out with the Le Bail approach.

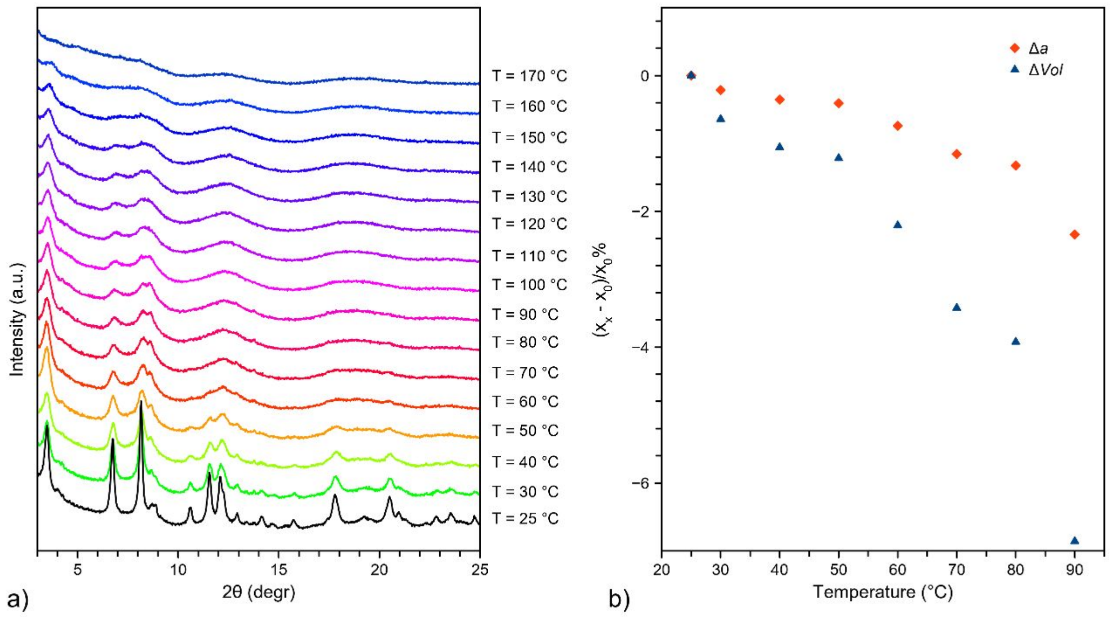

Figure S8. a) Powder X-ray diffraction patterns (Cu Kα, λ = 1.5418 Å) measured on as-synthesized **Zr-MOP-10** as a function of temperature heating in air, with steps of 10 °C, in the temperature range 25–170 °C. (b) Percentage variation of the unit cell parameters of **Zr-MOP-10** during the VT-PXRD experiment with respect to the T = 25 °C ones, as result of the parametric whole powder pattern refinement carried out with the Le Bail approach.

S.3.3. Single Cristal X-Ray Diffraction (SCXRD).

Single crystals of **Zr-MOP-1'**, **Zr-MOP-1-NH$_2$** and **Zr-MOP-10** could be obtained as detailed above. **Zr-MOP-1'** and **Zr-MOP-1-NH$_2$** data were collected at 130 K on a Bruker D8 VENTURE PHOTON III-14 diffractometer, employing graphite monochromatized Mo-Kα (λ = 0.71073 Å) radiation. **Zr-MOP-10** data were collected at 100 K with a Bruker D8 Venture diffractometer with graphite monochromated CuKα (λ = 1.54178 Å). Multi-scan absorption corrections were applied using the SADABS[2] routine. The structures were solved by direct methods with SHELXT-2014[3] and refined with SHELXL-2018[3] using OLEX 2-1.3.[4] All the non-hydrogen atoms were refined by full-matrix least-squares techniques. Due to positional disorder, in **Zr-MOP-1'** and **Zr-MOP-1-NH$_2$** some of the atoms of the *n*-butyl chain were modelled by splitting them into two positions. The same holds for one of the cyclopentadienyl rings in **Zr-MOP-1'**. Due to severe positional disorder, in **Zr-MOP-10** it was not possible to completely model the *n*-butyl chain. DFIX and SADI restrains were used to describe the *n*-butyl chains to obtain reasonable geometrical parameters, due to the observed disorder and the poor quality of the data. The carbon atoms of the *n*-butyl chains and of some of the cyclopentadienyl rings were not assigned anisotropic displacement parameters. In **Zr-MOP-1-NH$_2$** and **Zr-MOP-10**, the solvent molecules were found highly disordered and any attempts to describe the smeared electronic density belonging to them were unsuccessful. Contributions to diffraction due to these highly disordered molecules as well as the non-modelled *n*-butyl chains (see above) were removed using the SQUEEZE routine of PLATON;[5] the crystal structures were then refined again using the generated *hkl* data. When feasible, the hydrogen atoms were bound to their parent atoms in geometrically idealized positions through the so called riding model.

Final R_1 = 0.1062, wR_2 = 0.2193, $GOOF$ = 1.188, R_1 = 0.0741, wR_2 = 0.2058, $GOOF$ = 1.075, R_1 = 0.0965, wR_2 = 0.2814, $GOOF$ = 1.147, for **Zr-MOP-1'**, **Zr-MOP-1-NH$_2$** and **Zr-MOP-10**, respectively.

Crystal data and refinement results are shown in Table S1, while selected bond distances and angles are collected in Tables S2-S4. The CIF files have been deposited in the Cambridge Crystallographic Data Centre (CCDC) under deposition number 2153759 (**Zr-MOP-1'**), 2153760 (**Zr-MOP-1-NH$_2$**) and 2153911 (**Zr-MOP-10**).

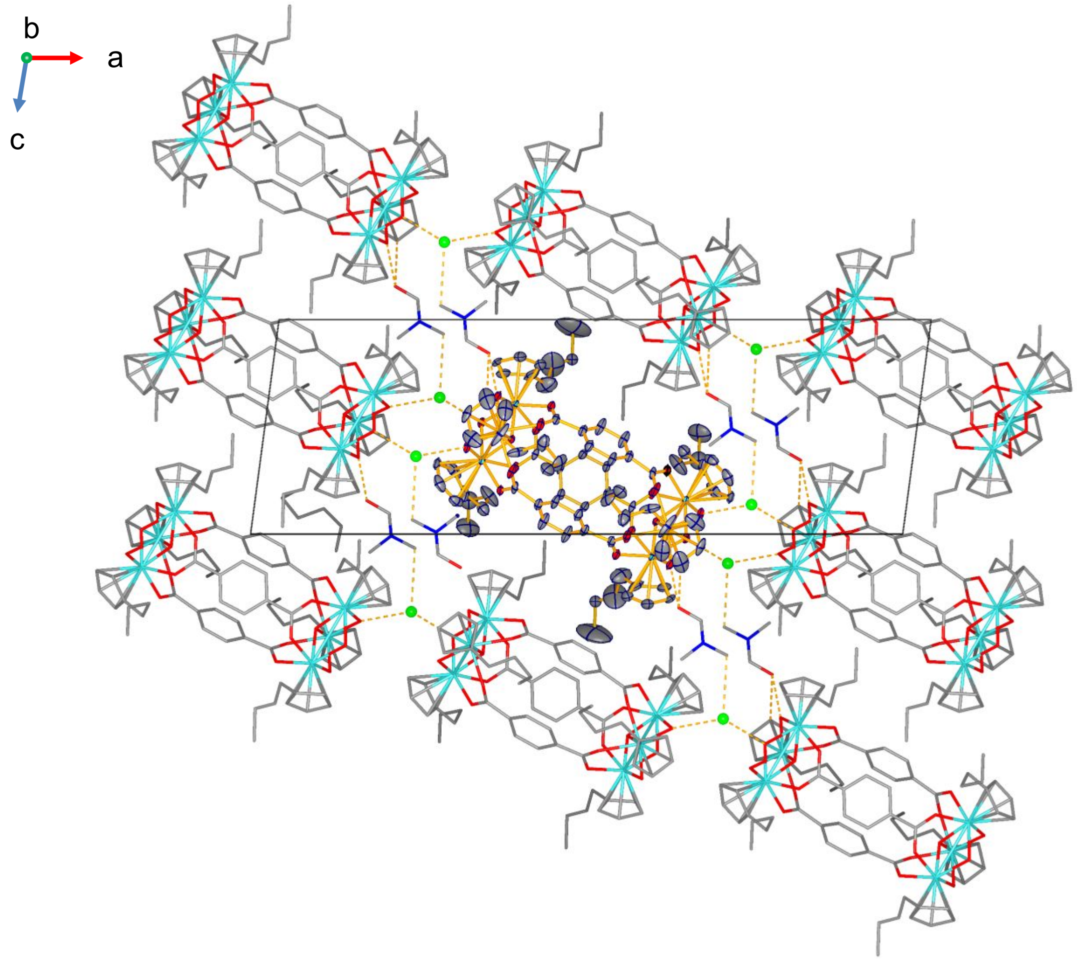

Figure S9. Portion of the crystal packing of **Zr-MOP-1'** viewed along the [010] crystallographic direction. Horizontal axis, *a*; vertical axis, *c*. Ellipsoids depicted with 50% probability level. Hydrogen atoms have been omitted for clarity. The non-bonding interactions O−H···Cl···H−O, C-H···Cl and O···H−O involving adjacent clusters and DMF are highlighted by dashed orange lines. Atom colour code: carbon, grey; chlorine, green; nitrogen, blue; oxygen, red; zirconium, light blue.

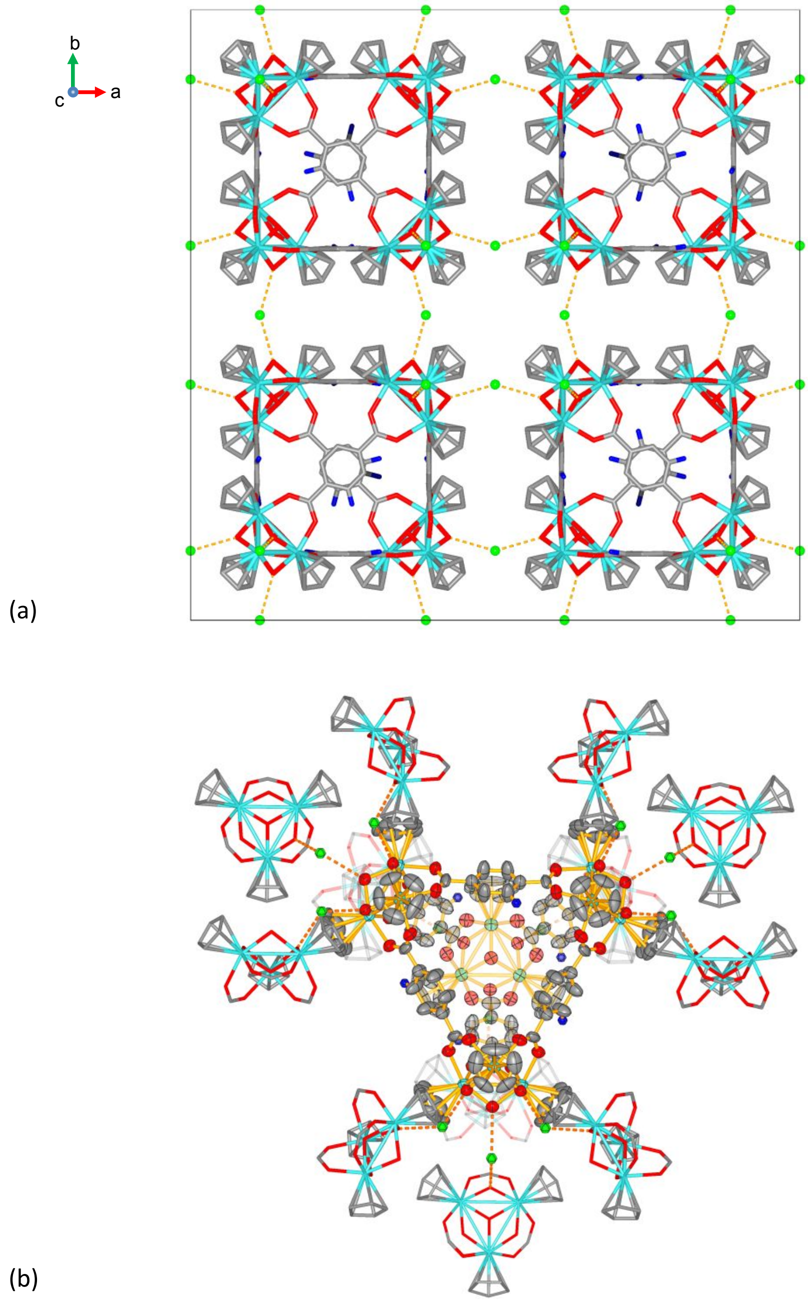

Figure S10. (a) Portion of the crystal packing of **Zr-MOP-1-NH$_2$** viewed along the [001] crystallographic direction. Horizontal axis, *a*; vertical axis, *b*. Ellipsoids depicted with 50% probability level. Hydrogen atoms and *n*-butyl chains have been omitted, while NH$_2$ groups have been ordered for clarity. (b) One tetrahedron. The non-bonding interactions O−H···Cl···H−O involving adjacent clusters are highlighted by dashed orange lines. Atom colour code: carbon, grey; chlorine, green; nitrogen, blue; oxygen, red; zirconium, light blue.

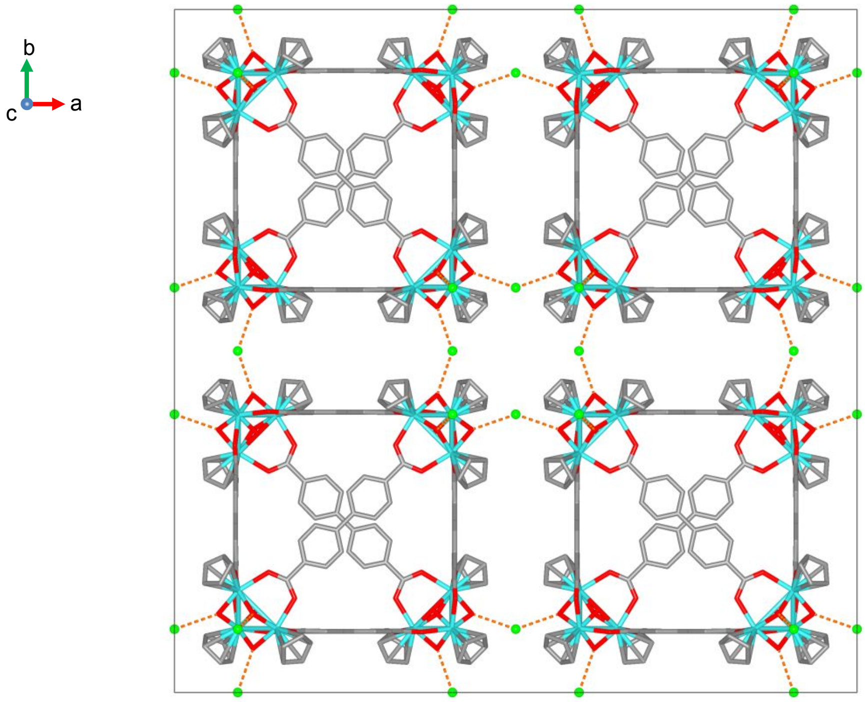

(a)

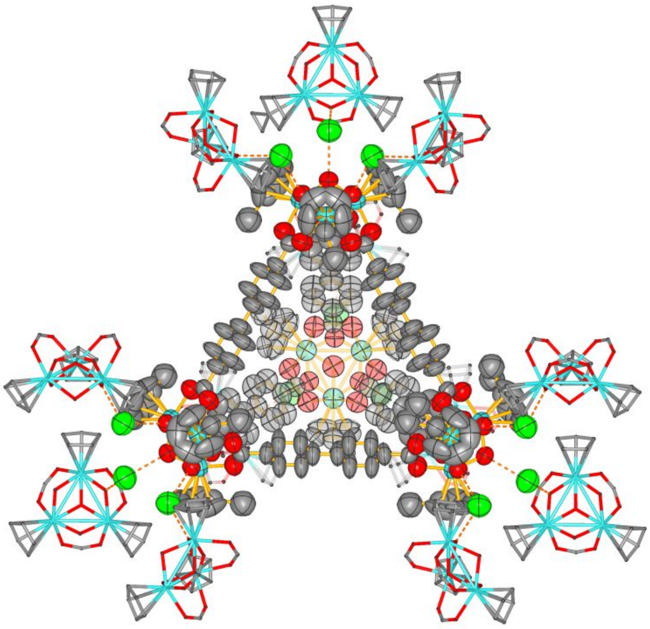

(b)

Figure S11. (a) Portion of the crystal packing of **Zr-MOP-10** viewed along the [001] crystallographic direction. Horizontal axis, *a*; vertical axis, *b*. Ellipsoids depicted with 50% probability level. Hydrogen atoms and *n*-butyl chains have been omitted, while the phenyl rings of the carboxylic ligand have been ordered for clarity. (b) One tetrahedron. The non-bonding interactions O−H···Cl···H−O involving adjacent clusters are highlighted by dashed orange lines. Atom colour code: carbon, grey; chlorine, green; oxygen, red; zirconium, light blue.

Table S1. Crystal data and refinement results for **Zr-MOP-1'**, **Zr-MOP-1-NH_2** and **Zr-MOP-10**.

	**Zr-MOP-1'**	**Zr-MOP-1-NH_2**	**Zr-MOP-10**
**Empirical formula**	$C_{84}H_{70}Cl_2N_2O_{22}Zr_6$	$C_{138}H_{48}Cl_6N_6O_{40}Zr_{12}$	$C_{156}H_{96}Cl_6O_{40}Zr_{12}$
**Formula weight/a.m.u.**	2077.64	3737.16	3917.66
**T/K**	130.0	130.0	100.0
**Crystal system**	monoclinic	cubic	cubic
**Space group**	*C*2/*c*	*Fm*-3*m*	*Fm*-3*m*
***a*/Å**	33.980(2)	36.777(3)	42.3762(5)
***b*/Å**	23.009(2)	36.777(3)	42.3762(5)
***c*/Å**	11.2581(8)	36.777(3)	42.3762(5)
***α*/°**	90	90	90
***β*/°**	97.560(3)	90	90
***γ*/°**	90	90	90
***V*/Å^3**	8725.6(12)	49743(12)	76097(2)
Z	4	8	8
**ρ_{calc} g/cm^3**	1.582	0.998	0.684
**μ/mm^{-1}**	0.822	0.591	3.230
***F*(000)**	4152.0	14560.0	15472.0
**Radiation**	MoKα<br>(λ = 0.71073)	MoKα<br>(λ = 0.71073)	CuKα<br>(λ = 1.54178)
**2Θ range for data collection/°**	4.288 to 57.58	4.828 to 41.688	3.612 to 72.638
**Index ranges**	-45 ≤ h ≤ 45<br>-31 ≤ k ≤ 31<br>-11 ≤ l ≤ 15	-20 ≤ h ≤ 36<br>-33 ≤ k ≤ 28<br>-36 ≤ l ≤ 16	-32 ≤ h ≤ 32<br>-32 ≤ k ≤ 32<br>-32 ≤ l ≤ 32
**Reflections collected**	101148	12096	55930
**Independent reflections**	11316<br>R_{int} = 0.0425,<br>R_σ = 0.0287	1351<br>R_{int} = 0.0542<br>R_σ = 0.0301	966<br>R_{int} = 0.2512<br>R_σ = 0.0251
**Data/restraints/parameters**	11316/13/543	1351/6/77	966/39/91
**Goodness-of-fit on F^2**	1.177	1.075	1.147
**Final R indexes [I≥2σ (I)]**	R_1 = 0.1077,<br>wR_2 = 0.2217	R_1 = 0.0741<br>wR_2 = 0.2058	R_1 = 0.0965<br>wR_2 = 0.2814
**Final R indexes [all data]**	R_1 = 0.1292,<br>wR_2 = 0.2343	R_1 = 0.0913<br>wR_2 = 0.2222	R_1 = 0.1344<br>wR_2 = 0.3484
**CCDC**	2153759	2153760	2153911

Table S2. Selected bond distances and angles of **Zr-MOP-1'**.

**Atoms**	**Length (Å)/Angle (°)**	**Atoms**	**Length (Å)/Angle (°)**
$Zr^{(1)}$ - $O^{(2)}$	2.132(5)	$O^{(2)}$ - $Zr^{(1)}$ - $C^{(32)}$	121.0(4)
$Zr^{(1)}$ - $O^{(3)1}$	2.103(6)	$O^{(2)}$ - $Zr^{(1)}$ - $C^{(33)}$	87.7(3)
$Zr^{(1)}$ - $O^{(4)}$	2.083(5)	$O^{(2)}$ - $Zr^{(1)}$ - $C^{(34)}$	78.7(3)
$Zr^{(1)}$ - $O^{(5)}$	2.220(6)	$O^{(2)}$ - $Zr^{(1)}$ - $C^{(35)}$	101.9(3)
$Zr^{(1)}$ - $C^{(31)}$	2.574(9)	$O^{(4)}$ - $Zr^{(1)}$ - $C^{(31)}$	153.9(3)
$Zr^{(1)}$ - $C^{(32)}$	2.519(10)	$O^{(4)}$ - $Zr^{(1)}$ - $C^{(32)}$	153.3(3)
$Zr^{(1)}$ - $C^{(33)}$	2.500(8)	$O^{(4)}$ - $Zr^{(1)}$ - $C^{(33)}$	151.2(3)
$Zr^{(1)}$ - $C^{(34)}$	2.532(11)	$O^{(4)}$ - $Zr^{(1)}$ - $C^{(34)}$	150.7(3)
$Zr^{(1)}$ - $C^{(35)}$	2.573(11)	$O^{(4)}$ - $Zr^{(1)}$ - $C^{(35)}$	152.2(3)
$Zr^{(2)}$ - $Zr^{(3)1}$	3.3450(14)	$O^{(5)}$ - $Zr^{(1)}$ - $C^{(31)}$	76.8(3)
$O^{(1)1}$ - $Zr^{(3)}$ - $O^{(8)}$	152.1(2)	$O^{(5)}$ - $Zr^{(1)}$ - $C^{(32)}$	88.1(4)
$O^{(1)1}$ - $Zr^{(3)}$ - $O^{(9)1}$	86.4(2)	$O^{(5)}$ - $Zr^{(1)}$ - $C^{(33)}$	121.5(3)
$O^{(2)}$ - $Zr^{(3)}$ - $O^{(8)}$	85.6(2)	$O^{(5)}$ - $Zr^{(1)}$ - $C^{(34)}$	127.8(3)
$O^{(4)}$ - $Zr^{(3)}$ - $O^{(2)}$	73.8(2)	$O^{(5)}$ - $Zr^{(1)}$ - $C^{(35)}$	100.0(3)
$O^{(4)}$ - $Zr^{(3)}$ - $O^{(8)}$	79.2(2)	$C^{(24)}$ - $Zr^{(2)}$ - $C^{(22)}$	53.5(4)
$O^{(2)}$ - $Zr^{(1)}$ - $C^{(31)}$	130.1(3)	$C^{(24)}$ – $Zr^{(2)}$ - $C^{(23)}$	32.1(5)
Symmetry transformations used to generate equivalent atoms: [1] -x, +y, 1/2-z			

Table S3. Selected bond distances and angles of **Zr-MOP-1-NH$_2$**.

**Atoms**	**Length (Å)/Angle (°)**	**Atoms**	**Length (Å)/Angle (°)**
$Zr^{(1)}$ - $O^{(1)1}$	2.131(5)	$O^{(1)}$ - $Zr^{(1)}$ - $O^{(3)}$	152.9(2)
$Zr^{(1)}$ - $O^{(2)}$	2.059(4)	$O^{(1)}$ - $Zr^{(1)}$ - $C^{(4)}$	79.4(3)
$Zr^{(1)}$ - $O^{(3)2}$	2.169(6)	$O^{(1)1}$ - $Zr^{(1)}$ - $C^{(4)}$	102.5(3)
$Zr^{(1)}$ - $Zr^{(1)1}$	3.335(2)	$O^{(1)1}$ - $Zr^{(1)}$ - $C^{(5)}$	121.6(3)
$Zr^{(1)}$ - $C^{(4)3}$	2.520(11)	$O^{(1)}$ - $Zr^{(1)}$ - $C^{(6)2}$	130.5(3)
$Zr^{(1)}$ - $C^{(5)}$	2.577(17)	$O^{(1)1}$ - $Zr^{(1)}$ - $C^{(6)2}$	92.0(3)
$Zr^{(1)}$ - $C^{(6)2}$	2.558(13)	$C^{(4)}$ - $Zr^{(1)}$ - $C^{(4)2}$	32.1(4)
$O^{(3)}$ - $C^{(1)}$	1.256(8)	$C^{(4)}$ - $Zr^{(1)}$ - $C^{(5)}$	50.0(4)
$O^{(1)}$ - $Zr^{(1)}$ - $O^{(1)1}$	92.6(4)	$C^{(4)2}$ - $Zr^{(1)}$ - $C^{(6)2}$	31.0(3)
$O^{(1)}$ - $Zr^{(1)}$ - $O^{(3)2}$	85.3(3)	$C^{(4)}$ - $Zr^{(1)}$ - $C^{(6)2}$	51.5(3)
Symmetry transformations used to generate equivalent atoms: [1] -x, +y, 1/2-z, [2] +x, +z, +y			

Table S4. Selected bond distances and angles of **Zr-MOP-10**.

**Atoms**	**Length (Å)/Angle (°)**	**Atoms**	**Length (Å)/Angle (°)**
$Zr^{(1)}$ - $O^{(1)2}$	2.125(11)	$O^{(1)}$ - $Zr^{(1)}$ - $O^{(3)}$	85.7(7)
$Zr^{(1)}$ - $O^{(2)}$	2.048(12)	$O^{(1)}$ - $Zr^{(1)}$ - $C^{(6)}$	79.1(8)
$Zr^{(1)}$ - $O^{(3)3}$	2.205(19)	$O^{(1)}$ - $Zr^{(1)}$ - $C^{(6)3}$	102.0(9)
$Zr^{(1)}$ - $Zr^{(1)1}$	3.344(6)	$O^{(1)}$ - $Zr^{(1)}$ - $C^{(8)}$	121.3(7)
$Zr^{(1)}$ - $C^{(6)3}$	2.45(3)	$O^{(1)}$ - $Zr^{(1)}$ - $C^{(7)3}$	129.9(9)
$Zr^{(1)}$ - $C^{(7)3}$	2.58(3)	$O^{(1)}$ - $Zr^{(1)}$ - $C^{(7)}$	90.5(9)
$Zr^{(1)}$ - $C^{(8)}$	2.50(4)	$C^{(6)3}$ - $Zr^{(1)}$ - $C^{(6)}$	31.8(16)
$O^{(3)}$ - $C^{(1)}$	1.24(2)	$C^{(6)3}$ - $Zr^{(1)}$ - $C^{(7)}$	51.5(9)
$O^{(1)}$ - $Zr^{(1)}$ - $O^{(1)2}$	92.3(10)	$C^{(6)3}$ - $Zr^{(1)}$ - $C^{(7)3}$	30.3(9)
$O^{(1)}$ - $Zr^{(1)}$ - $O^{(3)3}$	153.9(6)	$C^{(6)}$ - $Zr^{(1)}$ - $C^{(8)}$	49.7(13)
Symmetry transformations used to generate equivalent atoms: [1] 1/2 -z, +x, 1/2-y; [2] +y, 1/2-z, 1/2-x; [3] 1/2-z, +y, 1/2-x; [4] +x, 1/2-z, 1/2-y; [5] +x, 1/2-y, 1/2-z			

S.3.4. ^{1}H Nuclear Magnetic Resonance Spectroscopy.

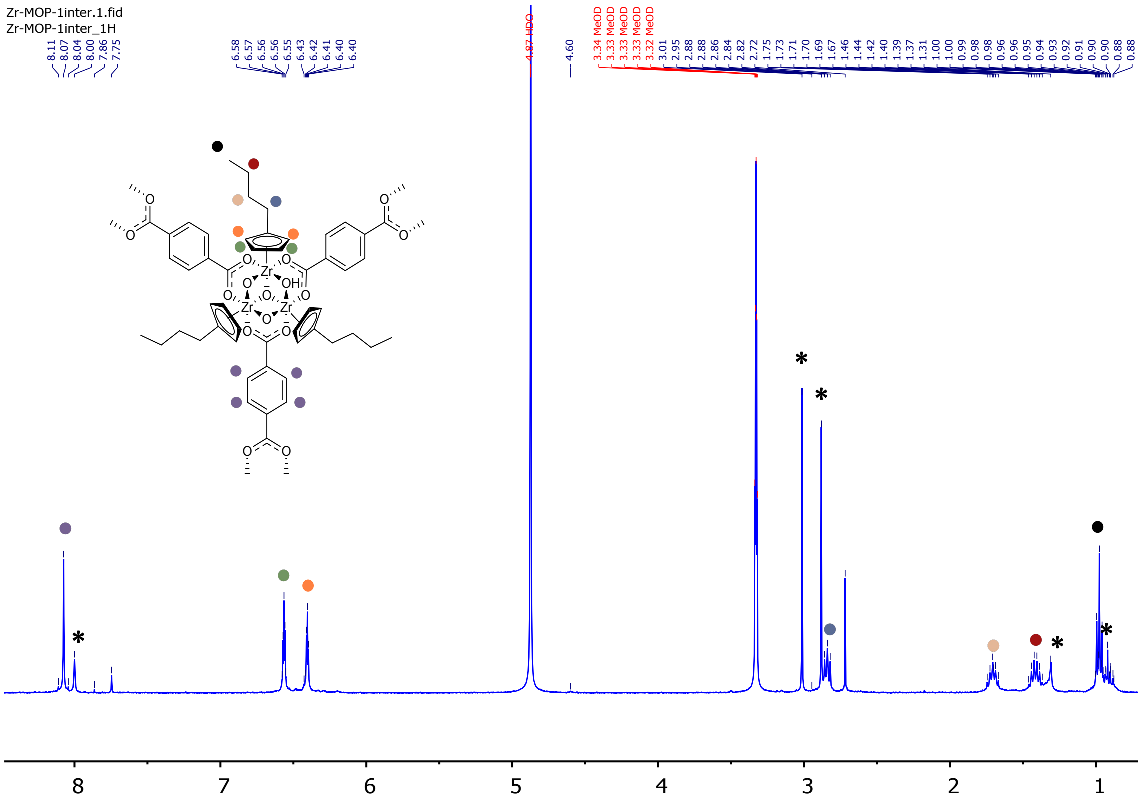

Figure S12. ^{1}H NMR spectra of a **Zr-MOP-1** solution in CD_3OD. The signals marked with an asterisk correspond to the solvents N,N-dimethylformamide and *n*-hexane adsorbed on the porous structure of the material.

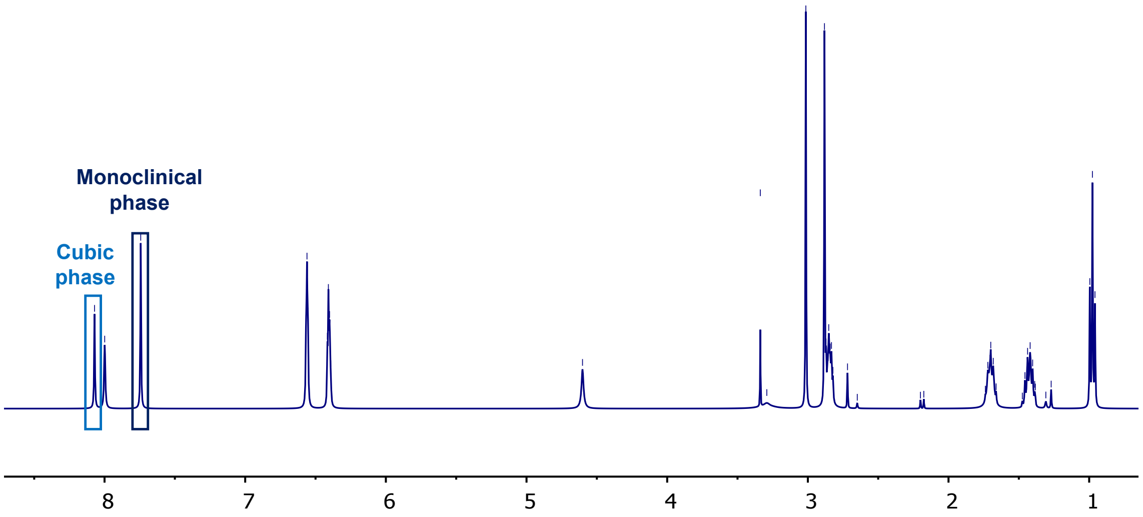

Figure S13. ^{1}H NMR spectra of a **Zr-MOP-1’** solution in CD_3OD.

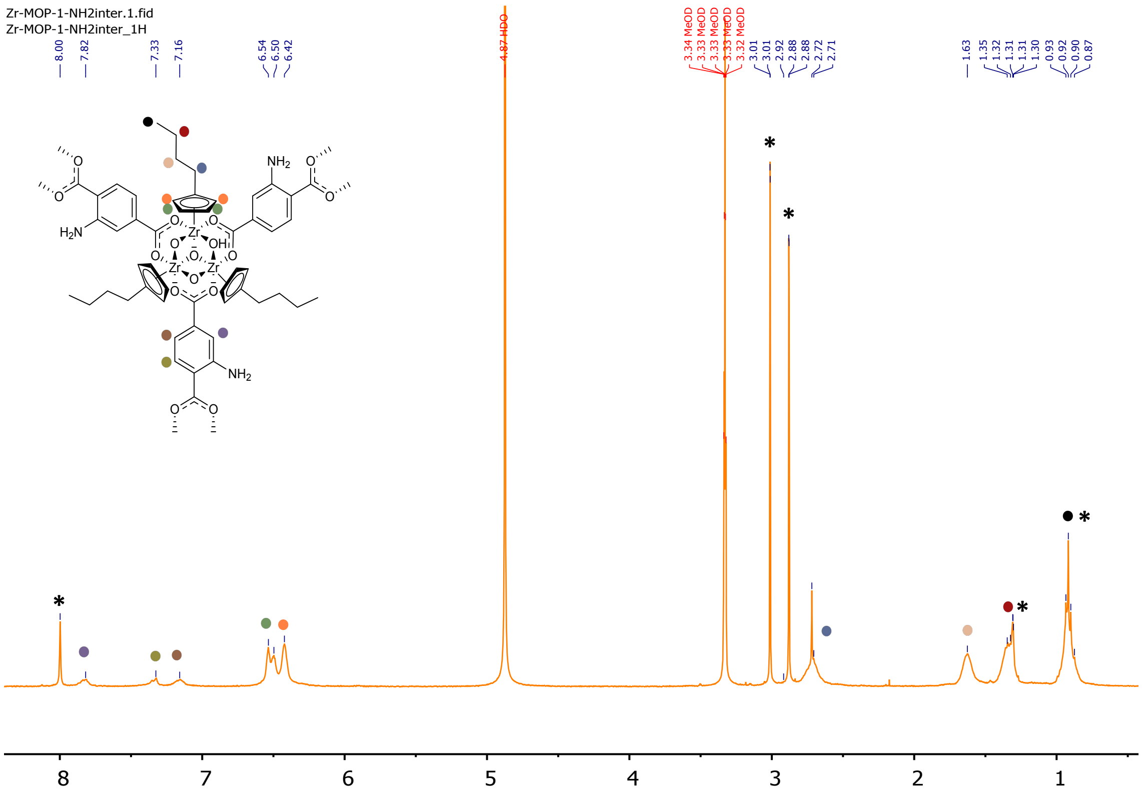

Figure S14. ^{1}H NMR spectra of a **Zr-MOP-1-NH$_2$** solution in CD_3OD. The signals marked with an asterisk correspond to the solvents N,N-dimethylformamide and *n*-hexane adsorbed on the porous structure of the material.

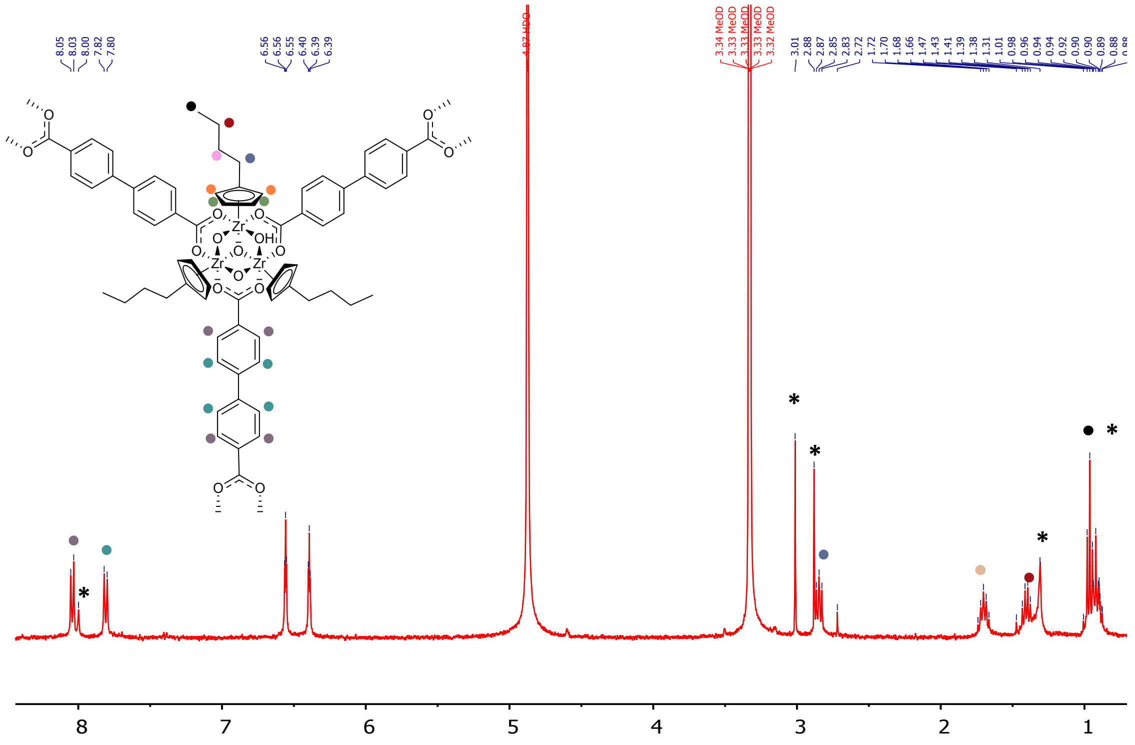

Figure S15. ^{1}H NMR spectra of a **Zr-MOP-10** solution in CD_3OD. The signals marked with an asterisk correspond to the solvents N,N-dimethylformamide and *n*-hexane adsorbed on the porous structure of the material.

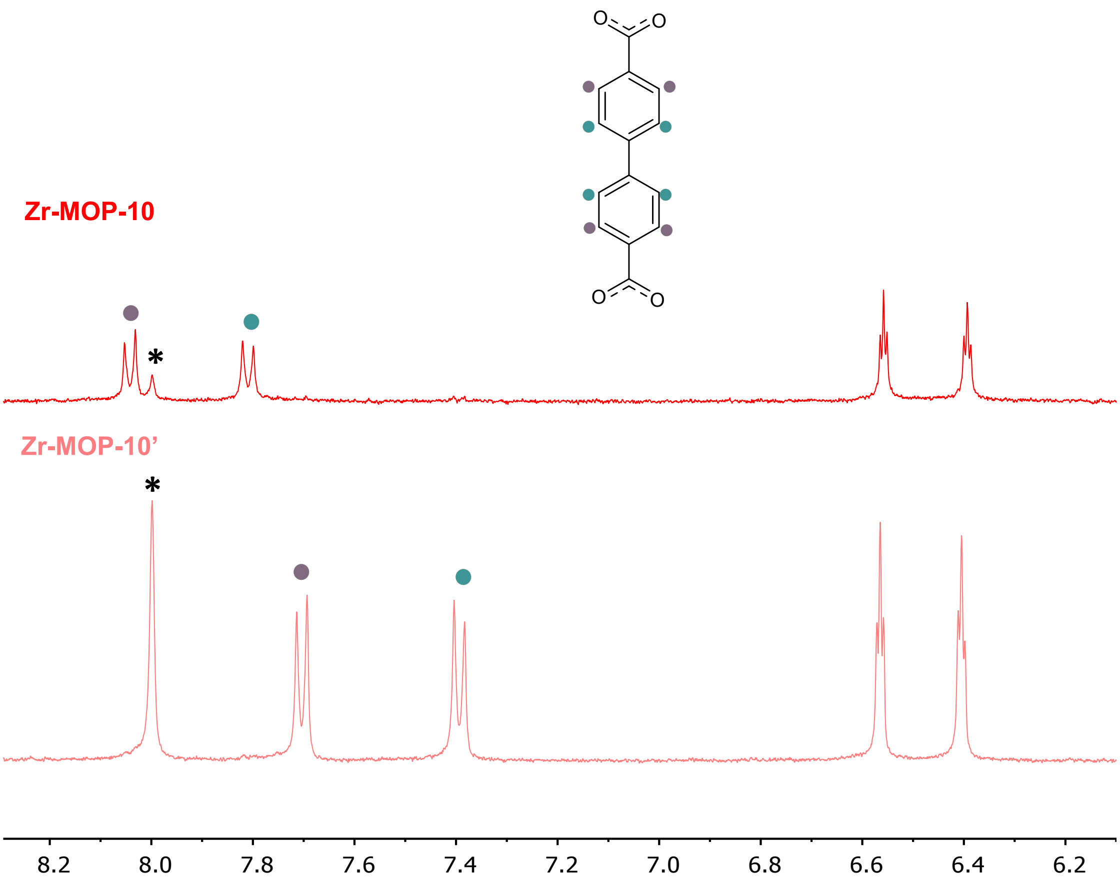

Figure S16. Comparison of the aromatic zone of the ^{1}H NMR spectra of a **Zr-MOP-10** and **Zr-MOP-10'** solution in CD_3OD. The signals marked with an asterisk correspond to the solvent N,N-dimethylformamide adsorbed on the porous structure of the material.

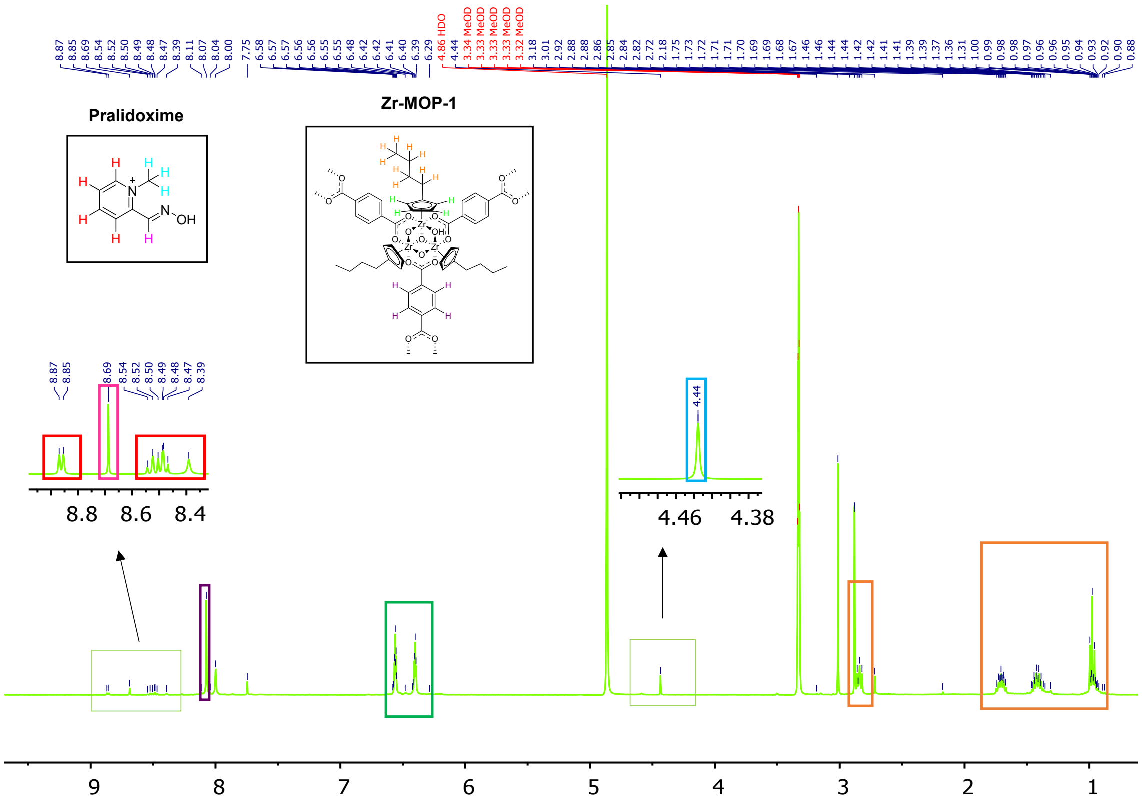

Figure S17. ^{1}H NMR spectrum of a solution of **Zr-MOP-1@2-PAM** in CD_3OD.

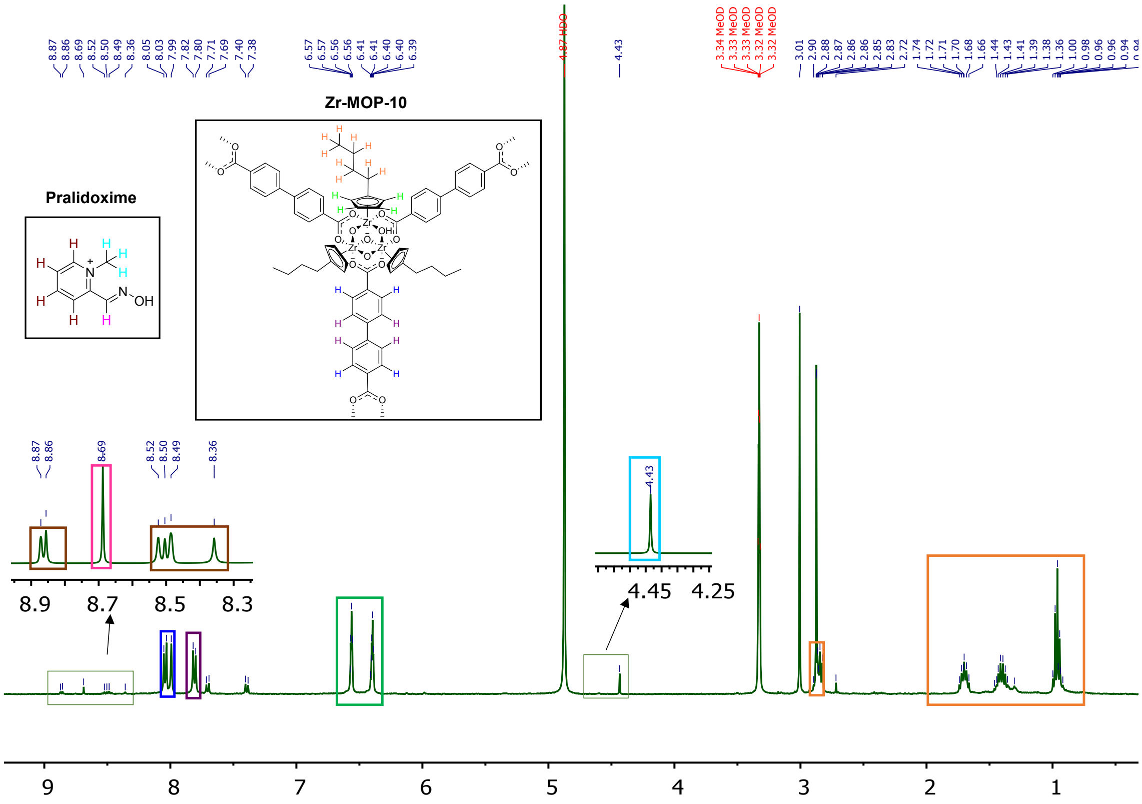

Figure S18. ^{1}H NMR spectrum of a solution of **Zr-MOP-10@2-PAM** in CD_3OD.

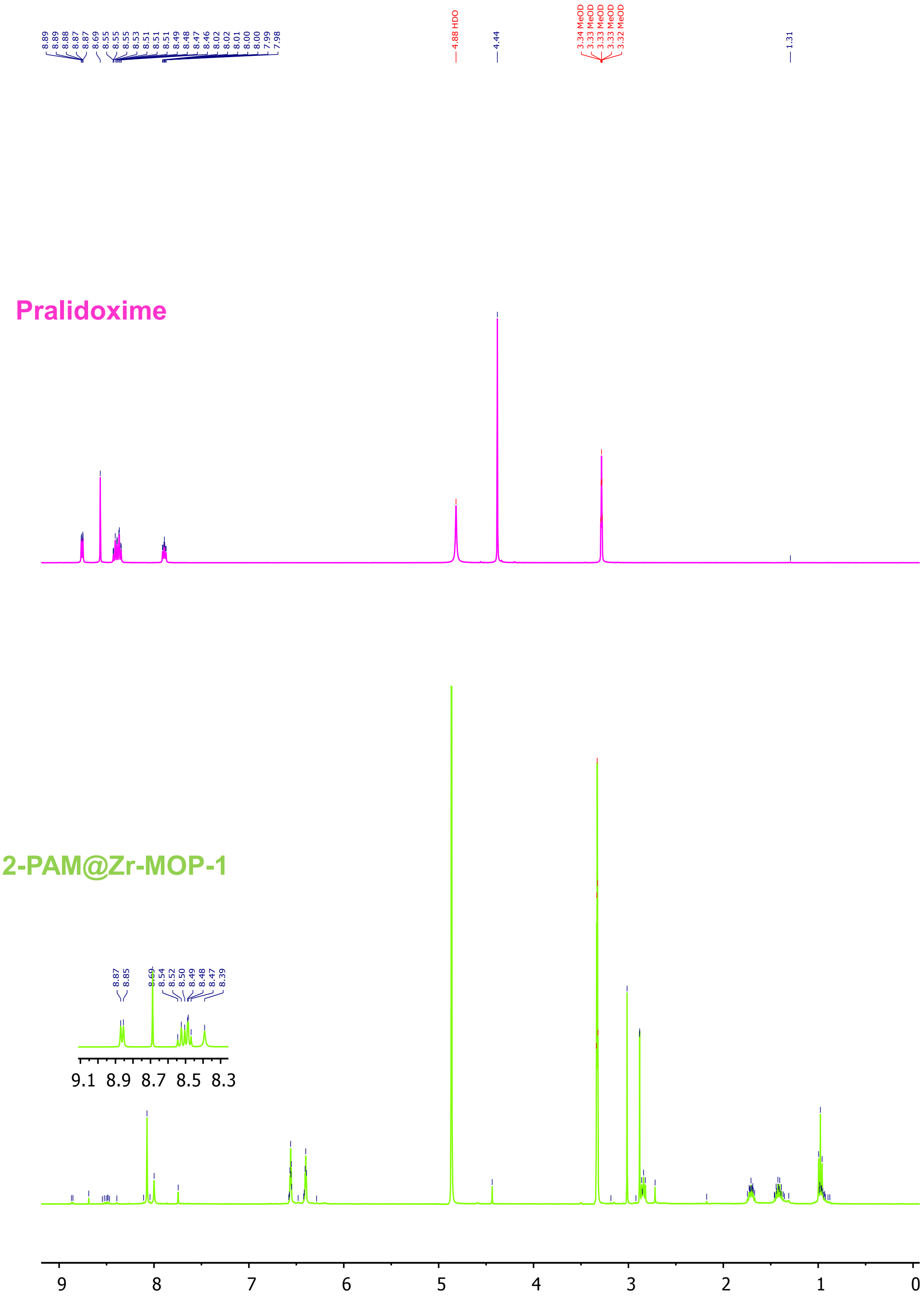

Figure S19. Comparison of the ^{1}H NMR spectra of pralidoxime (top) and **Zr-MOP-1@2-PAM** (bottom) in CD_3OD.

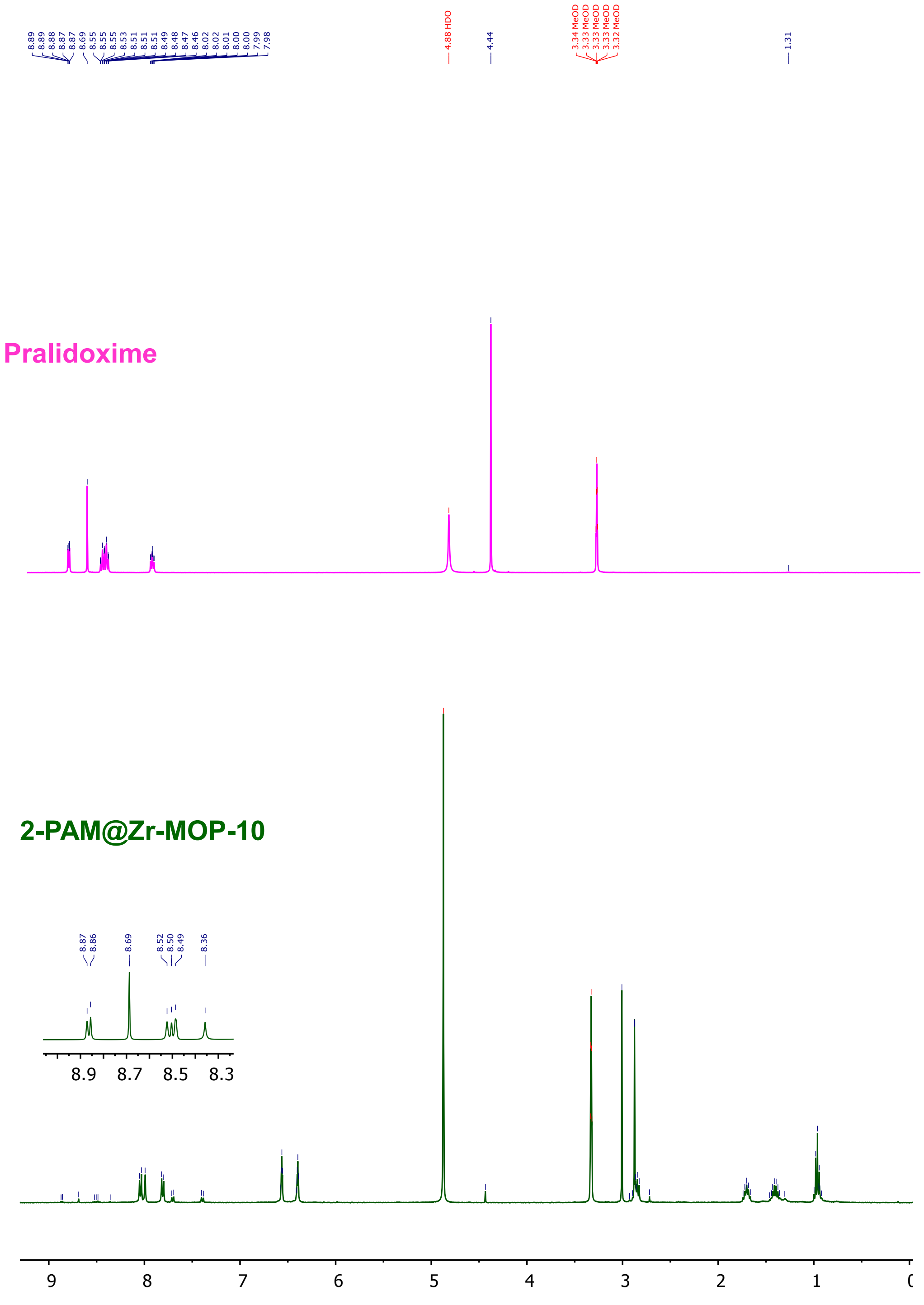

Figure S20. Comparison of the ^{1}H NMR spectra of pralidoxime (top) and **Zr-MOP-10@2-PAM** (bottom) in CD_3OD.

S.3.5. High resolution mass spectrometry (ESI-MS).

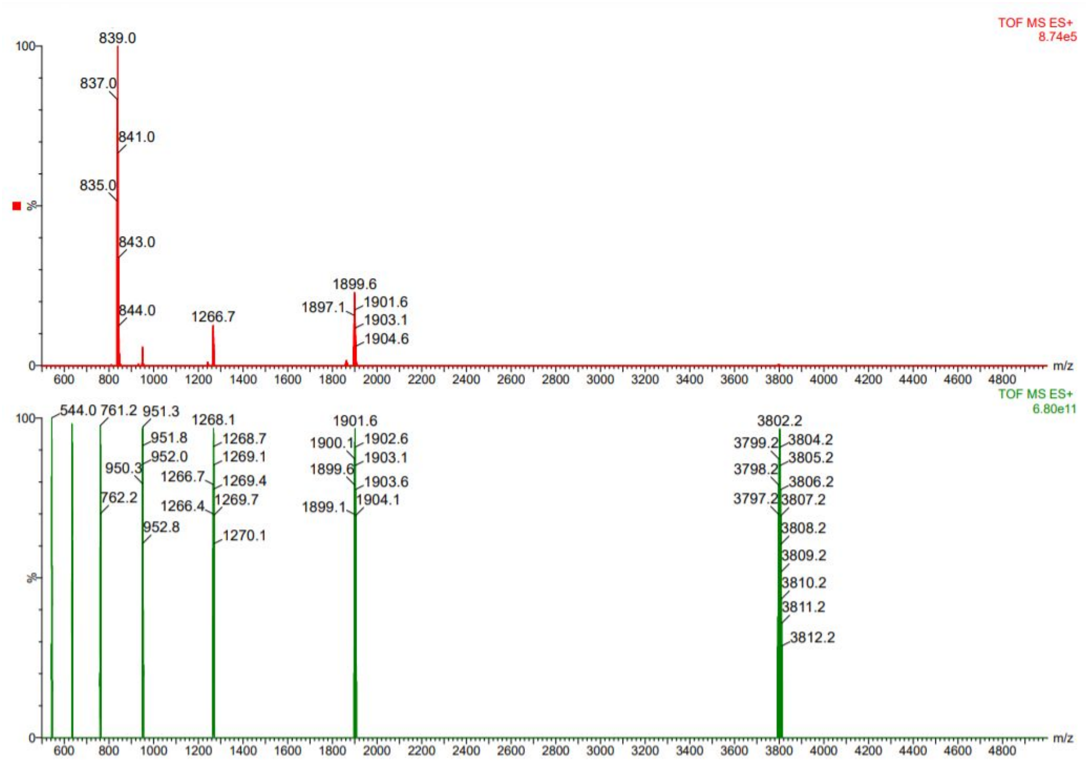

Figure S21. High-resolution ESI-MS spectrum of **Zr-MOP-1**. Experimental (top) and calculated (bottom) ESI-MS spectrum.

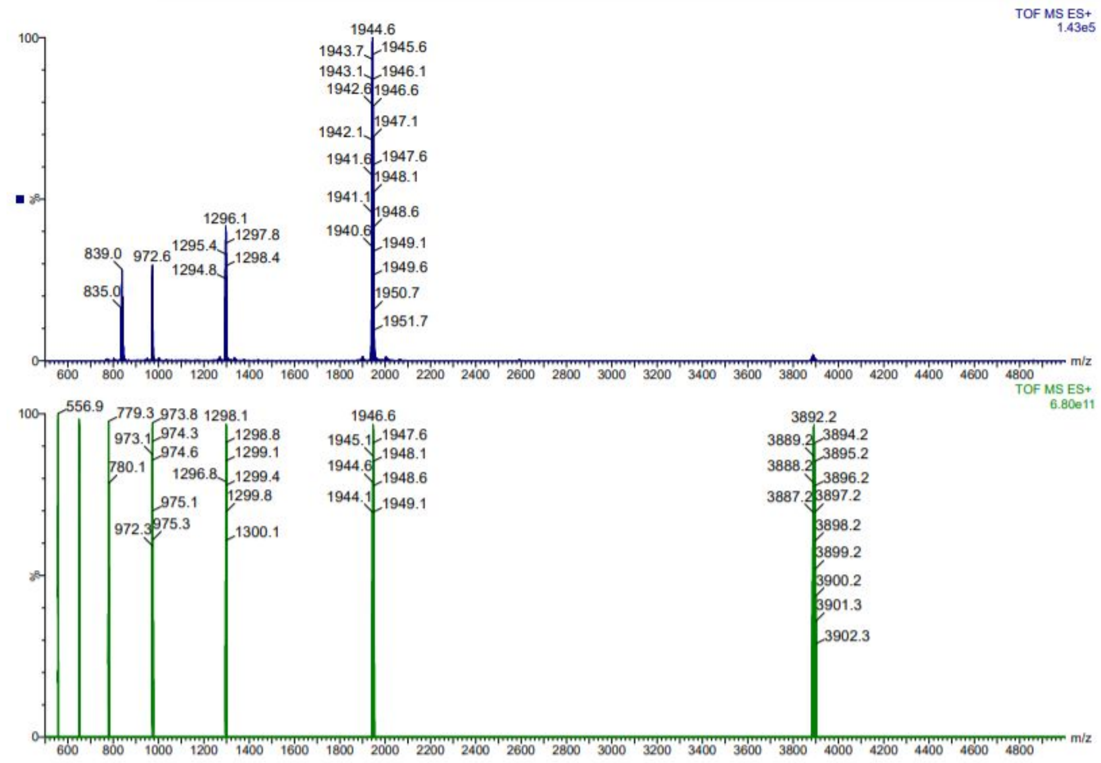

Figure S22. High-resolution ESI-MS spectrum of **Zr-MOP-1-NH$_2$**. Experimental (top) and calculated (bottom) ESI-MS spectrum.

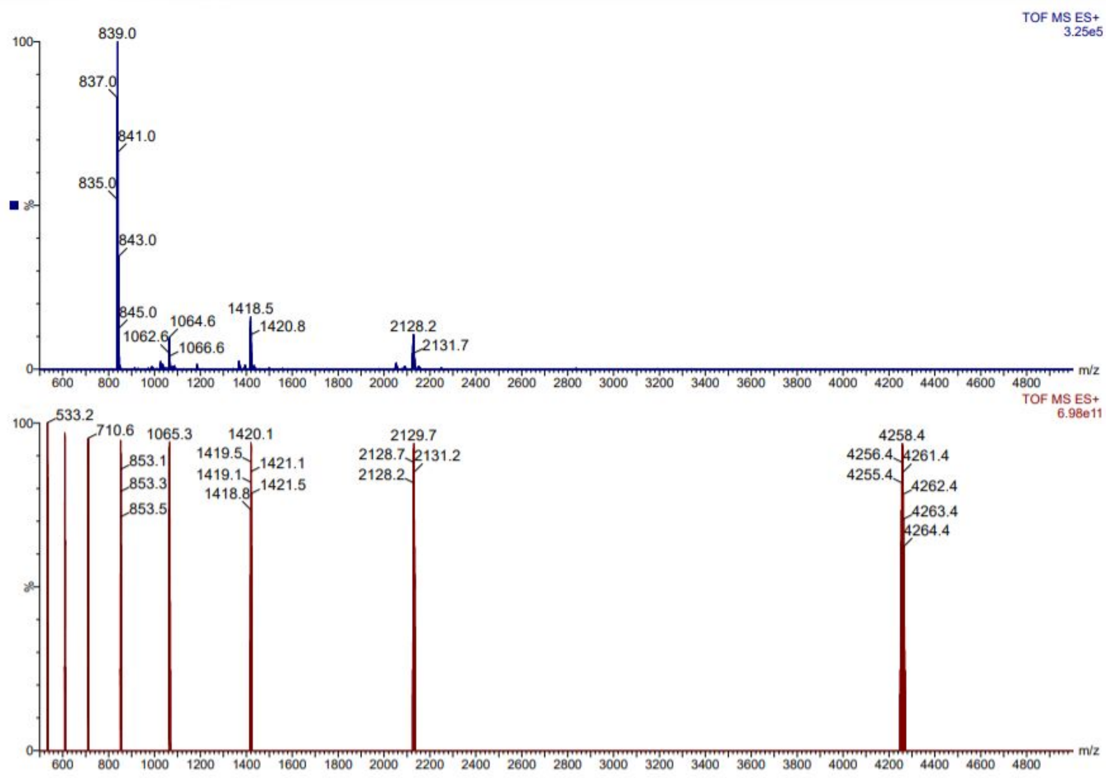

Figure S23. High-resolution ESI-MS spectrum of **Zr-MOP-10**. Experimental (top) and calculated (bottom) ESI-MS spectrum.

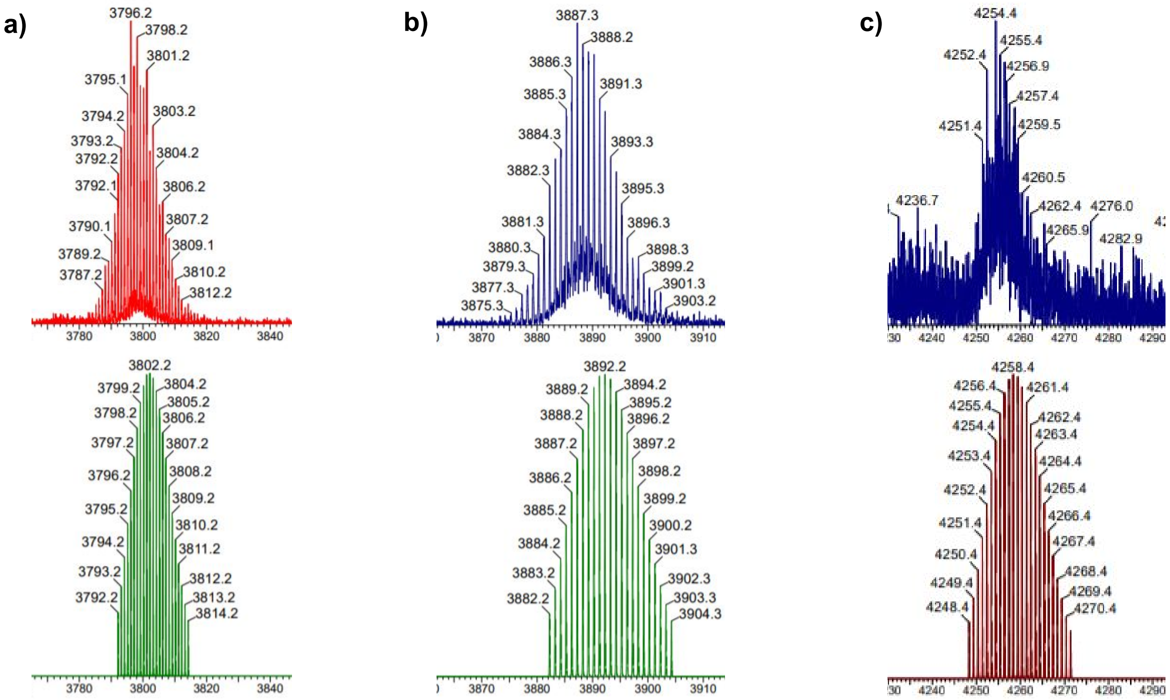

Figure S24. Magnification of the high-resolution ESI-MS spectrum: peaks of $[Zr\text{-}MOP\text{-}1\text{-}2Cl\text{-}6H]^+$ (a), $[Zr\text{-}MOP\text{-}1\text{-}NH_2\text{-}2Cl\text{-}6H]^+$ (b), $[Zr\text{-}MOP\text{-}10\text{-}2Cl\text{-}6H]^+$ (c). Experimental (top) and calculated (bottom) ESI-MS spectrum.

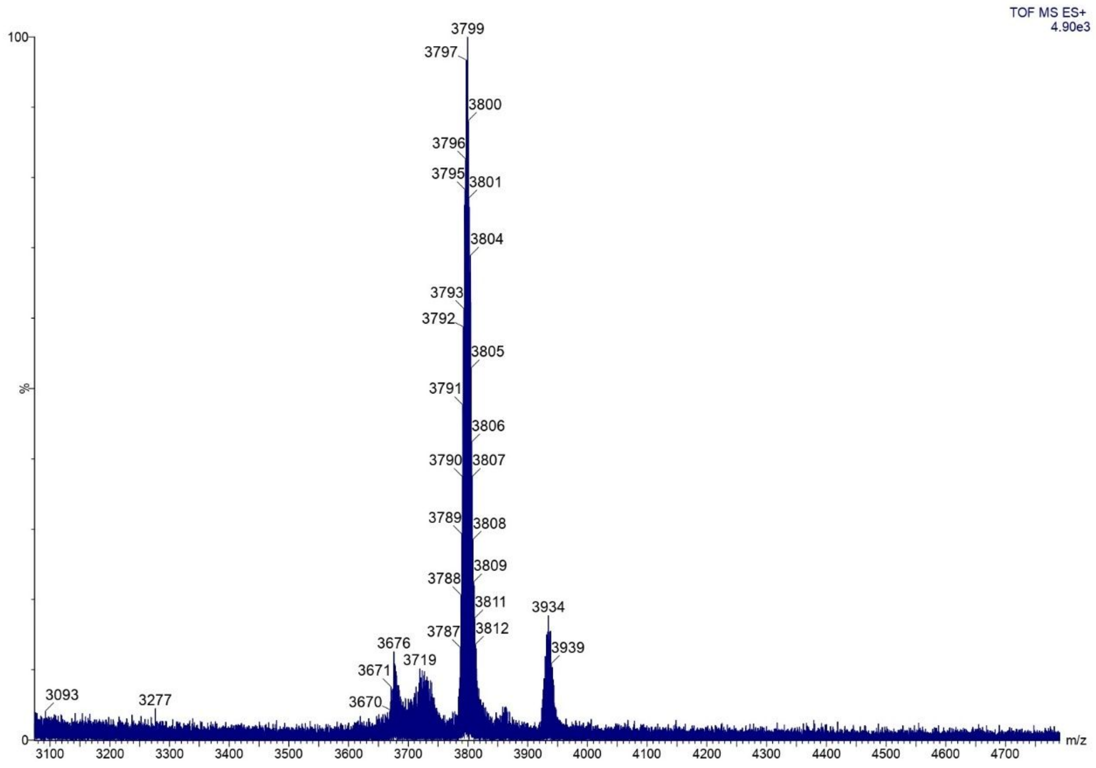

Figure S25. High-resolution ESI-MS spectrum of **Zr-MOP-1@2-PAM**. The higher intensity peak corresponds to [Zr-MOP-1-2Cl-6H]$^{+}$ (3799 m/z) and the second one to [Zr-MOP-1-2Cl-6H+2-PAM]$^{+}$ (3934 m/z).

S.3.6. Thermogravimetric Analysis (TGA) and Differential Scanning Calorimetry (DSC).

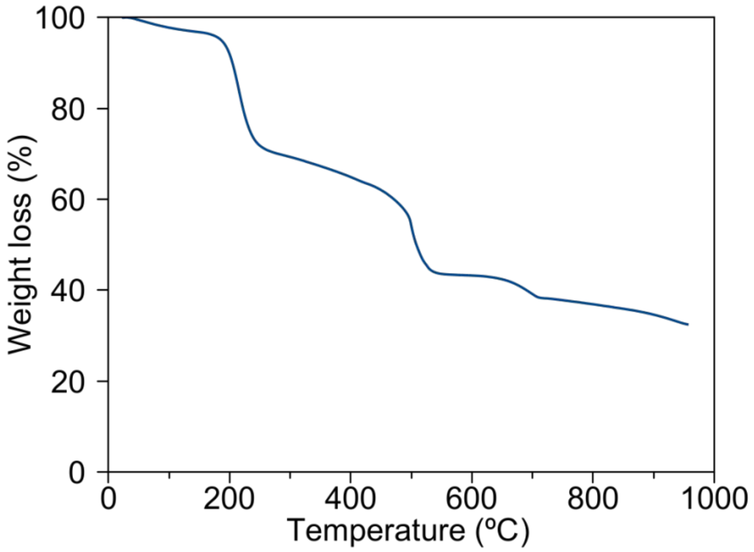

Figure S26. Thermogravimetric analysis of **Zr-MOP-1**.

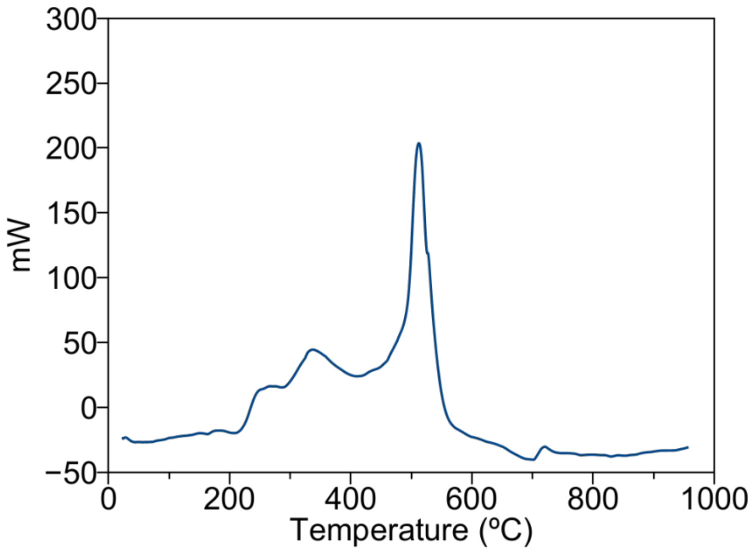

Figure S27. Differential scanning calorimetry of **Zr-MOP-1**.

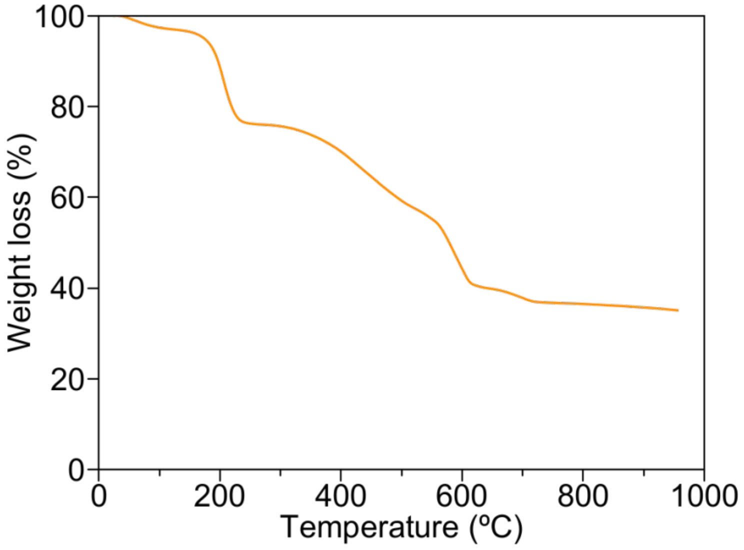

Figure S28. Thermogravimetric analysis of **Zr-MOP-1-NH$_2$**.

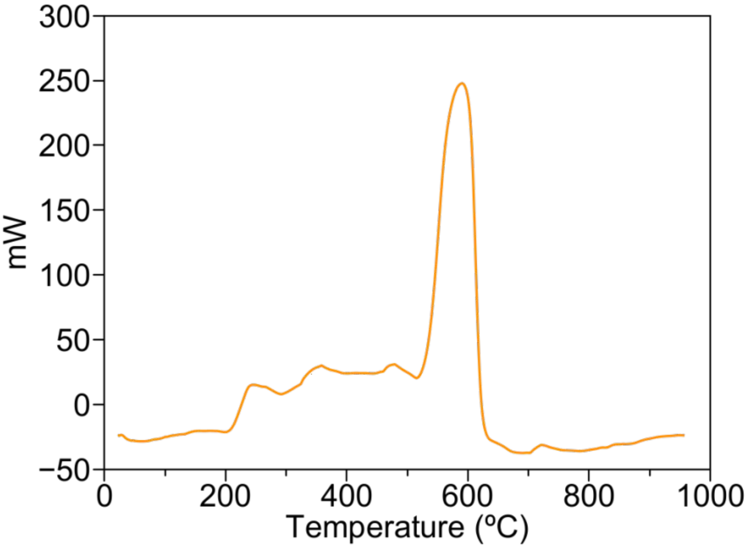

Figure S29. Differential scanning calorimetry of **Zr-MOP-1-NH$_2$**.

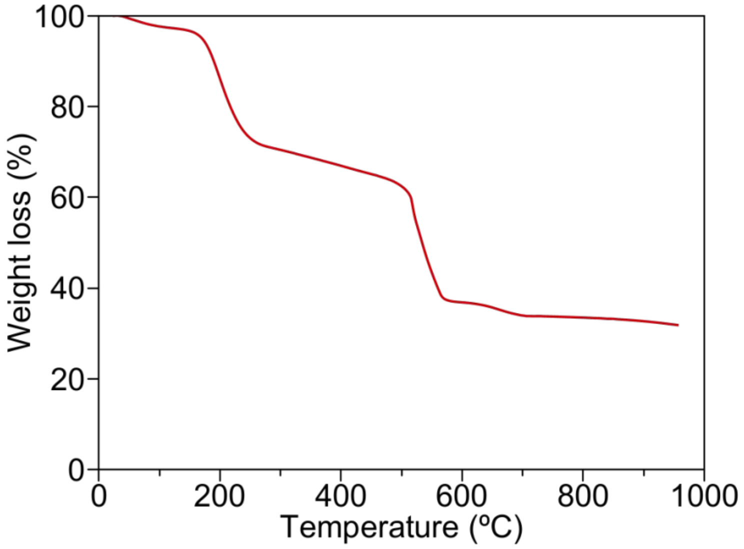

Figure S30. Thermogravimetric analysis of **Zr-MOP-10**.

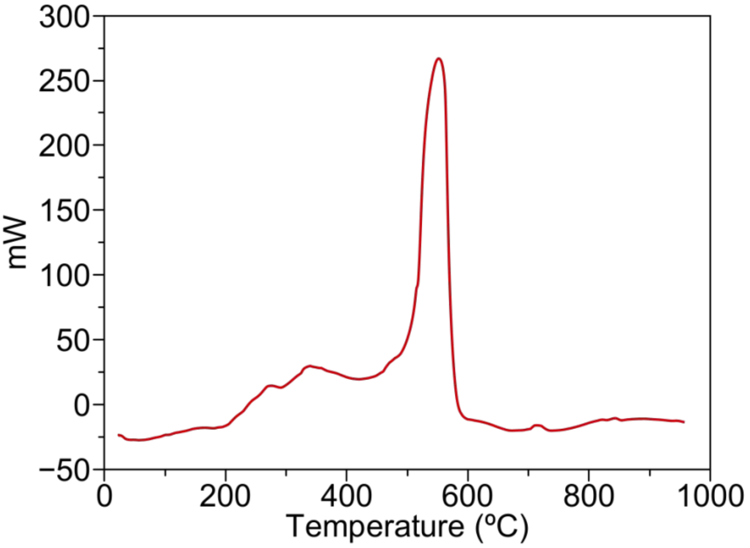

Figure S31. Differential scanning calorimetry of **Zr-MOP-10**.

S.3.7. Gas adsorption isotherms.

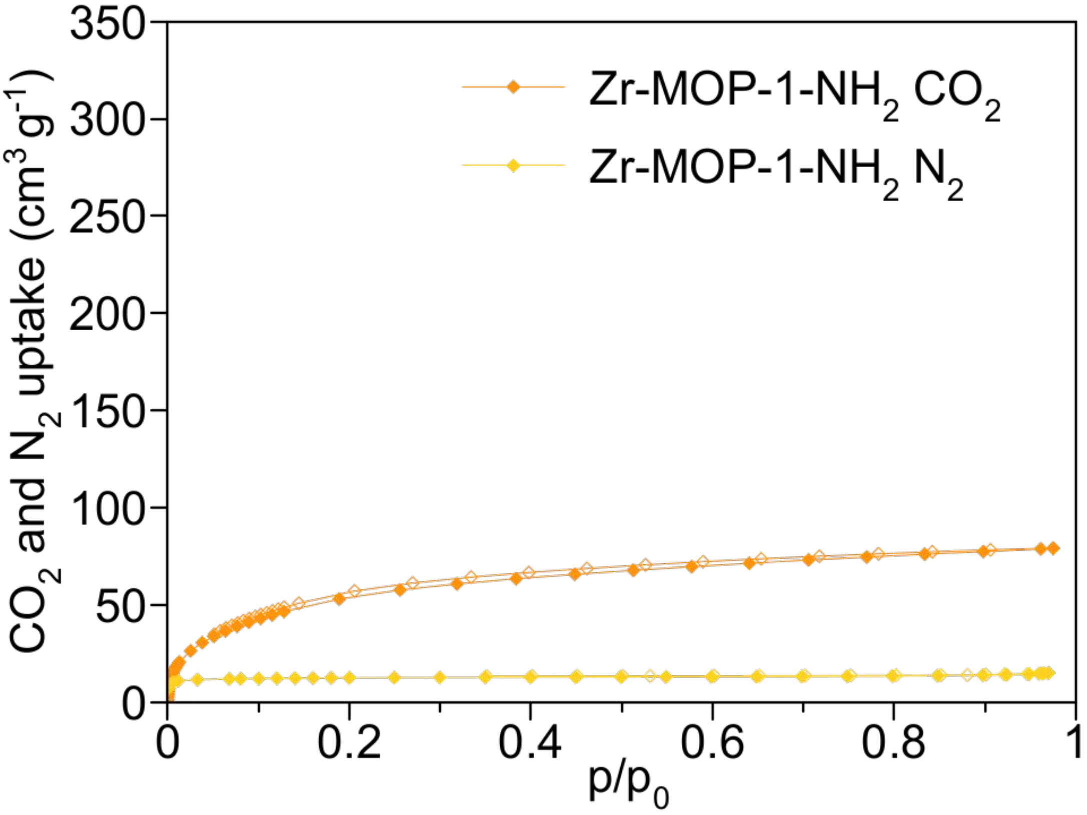

Figure S32. Nitrogen adsorption isotherm of **Zr-MOP-1-NH$_2$** (yellow) at 77 K and CO_2 adsorption isotherm of **Zr-MOP-1-NH$_2$** (orange) at 195 K. BET surface area for N_2 adsorption: almost 0. BET surface area for CO_2 (195 K) adsorption: 177 m^2/g.

S.3.8. Fourier Transform Infrared Spectroscopy (FTIR).

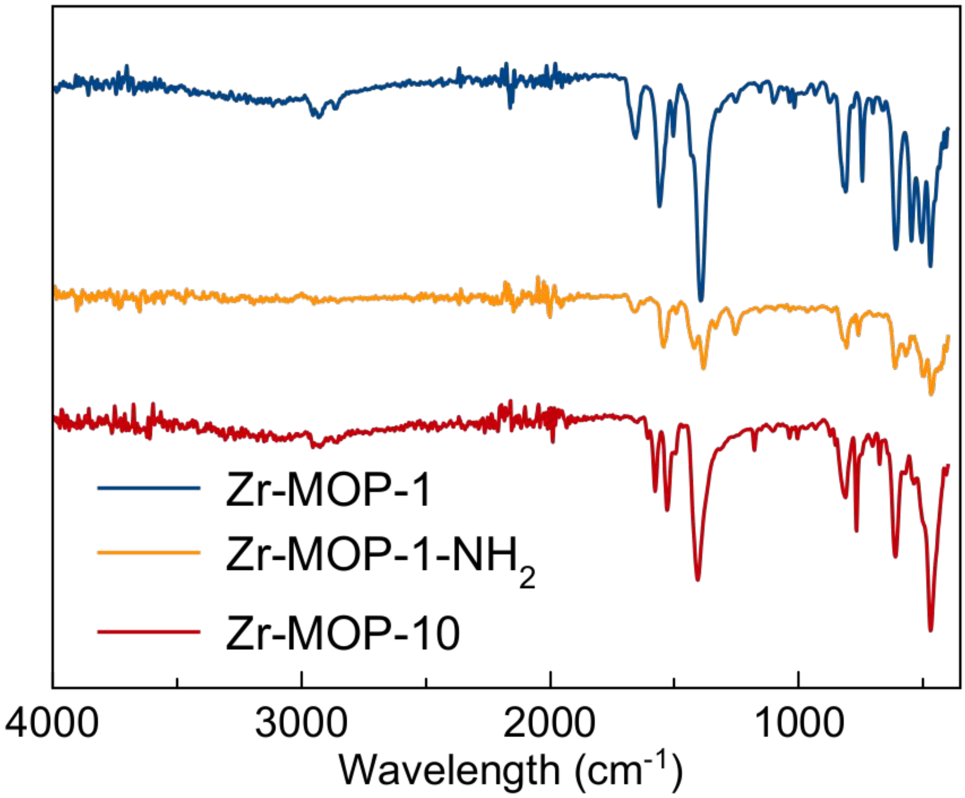

Figure S33. FTIR of **Zr-MOP-1** (blue), **Zr-MOP-1-NH$_2$** (yellow) and **Zr-MOP-10** (red).

S.3.9. Scanning Electron Microscopy–Energy Dispersive X-Ray (SEM-EDX).

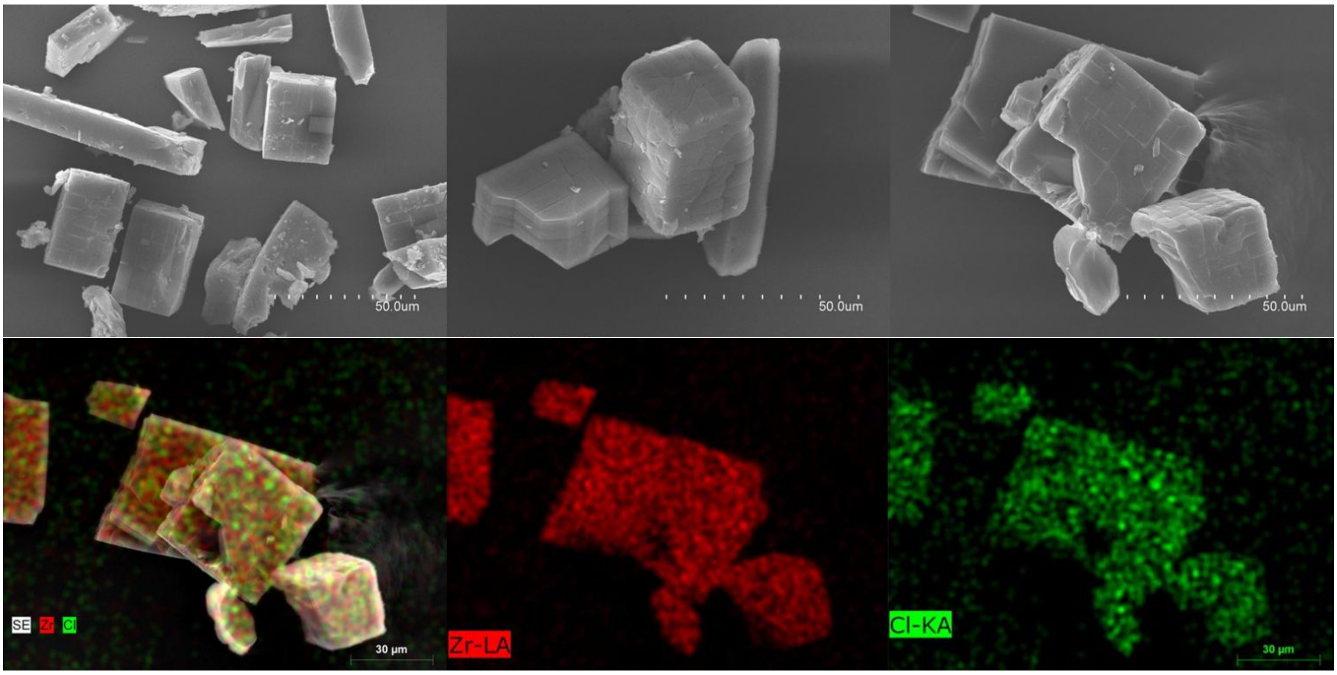

Figure S34. SEM-EDX images of **Zr-MOP-1**.

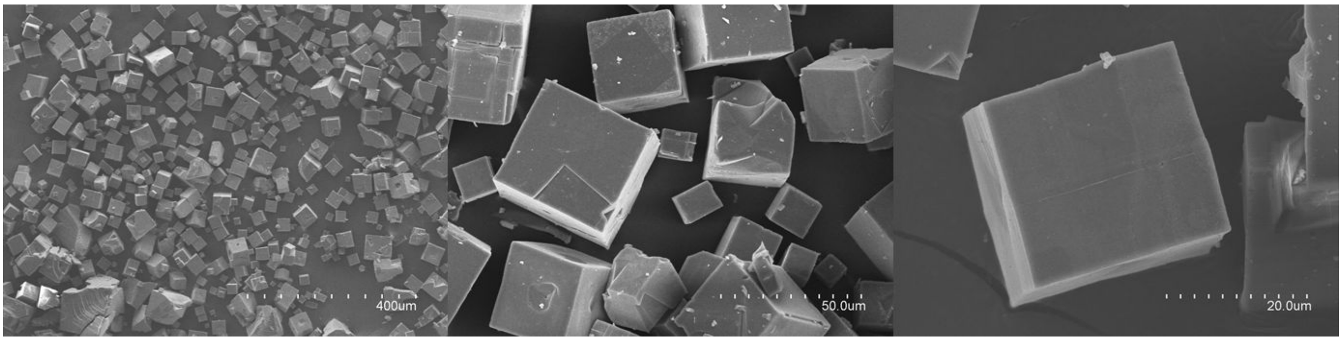

Figure S35. SEM images of **Zr-MOP-1-NH_2**.

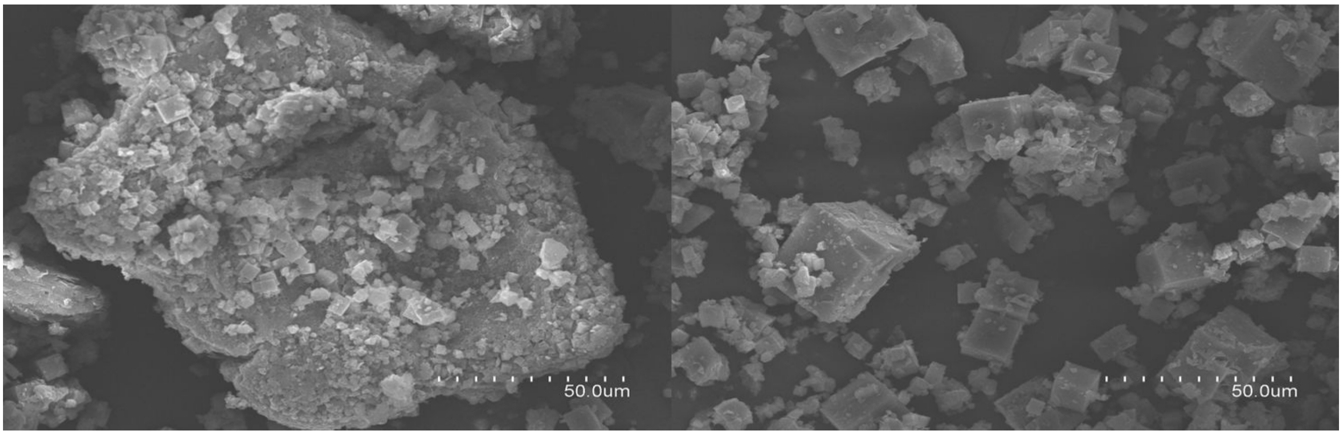

Figure S36. SEM images of **Zr-MOP-10**.

S.4. DIFP adsorption studies.

S.4.1. Gas cromatography studies.

We proceeded to study the adsorption of diisopropylfluorophosphate (DIFP) as a model of the nerve agent (CWA). The adsorption of DIFP was studied adding 10 mg of **Zr-MOP-1**, **Zr-MOP-1-NH$_2$** and **Zr-MOP-10**, 500 µL of H_2O, 1.25 µL of DMA and 1.25 µL of DIFP in a closed vial with a septum. The concentration of DIFP in the supernatant was measured at different time lapses.

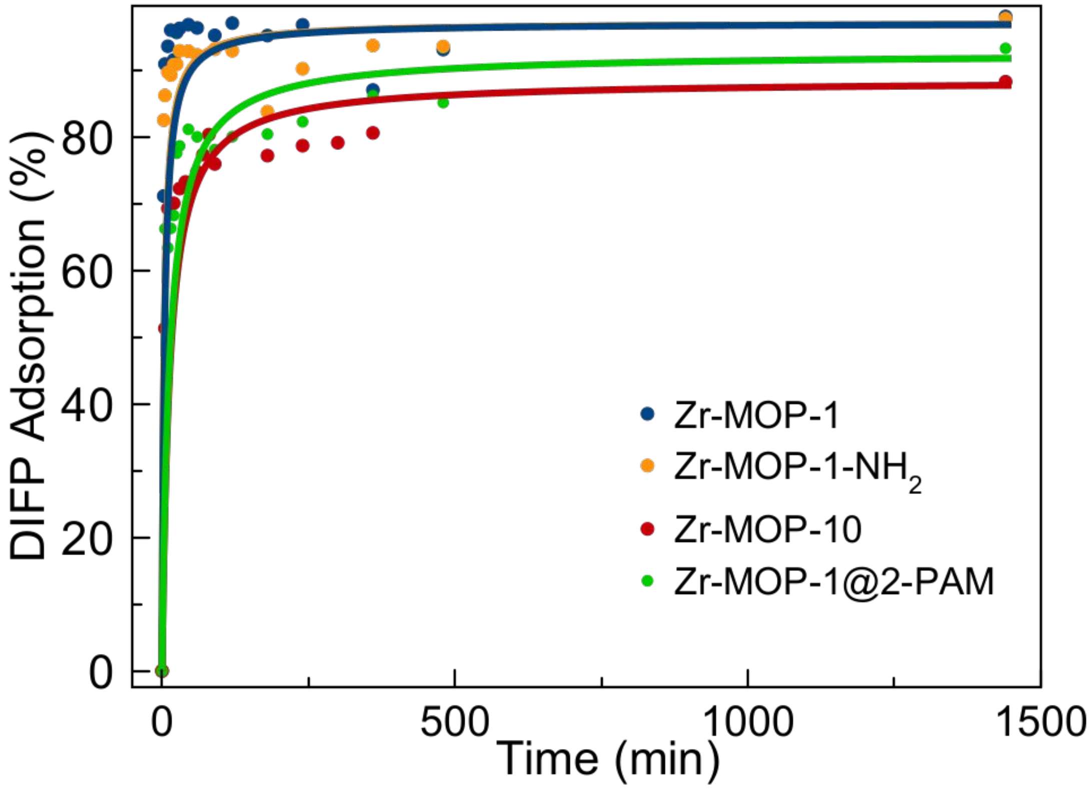

Figure S37. DIFP capture profiles of **Zr-MOP-1**, **Zr-MOP-1-NH$_2$**, **Zr-MOP-10** and **Zr-MOP-1@2-PAM** from an unbuffered aqueous suspension with a 1:2 MOP:DIFP ratio. The curves correspond to pseudo-second order adsorption kinetic model.

S.4.2. ^{31}P NMR studies.

The adsorption study of DIFP by the different MOPs, namely **Zr-MOP-1**, **Zr-MOP-1-NH$_2$** and **Zr-MOP-10**, was also studied by ^{31}P NMR. In a typical experiment, 6 mg of each material were mixed with 0.5 mL of D_2O and 0.6 µL of DIFP. After 15 min or 24 h, the supernatant was separated from the solid by centrifugation. The solid was suspended in CD_3OD to extract the compounds trapped inside the cavities. Finally, both fraction were analysed by ^{31}P NMR spectroscopy.

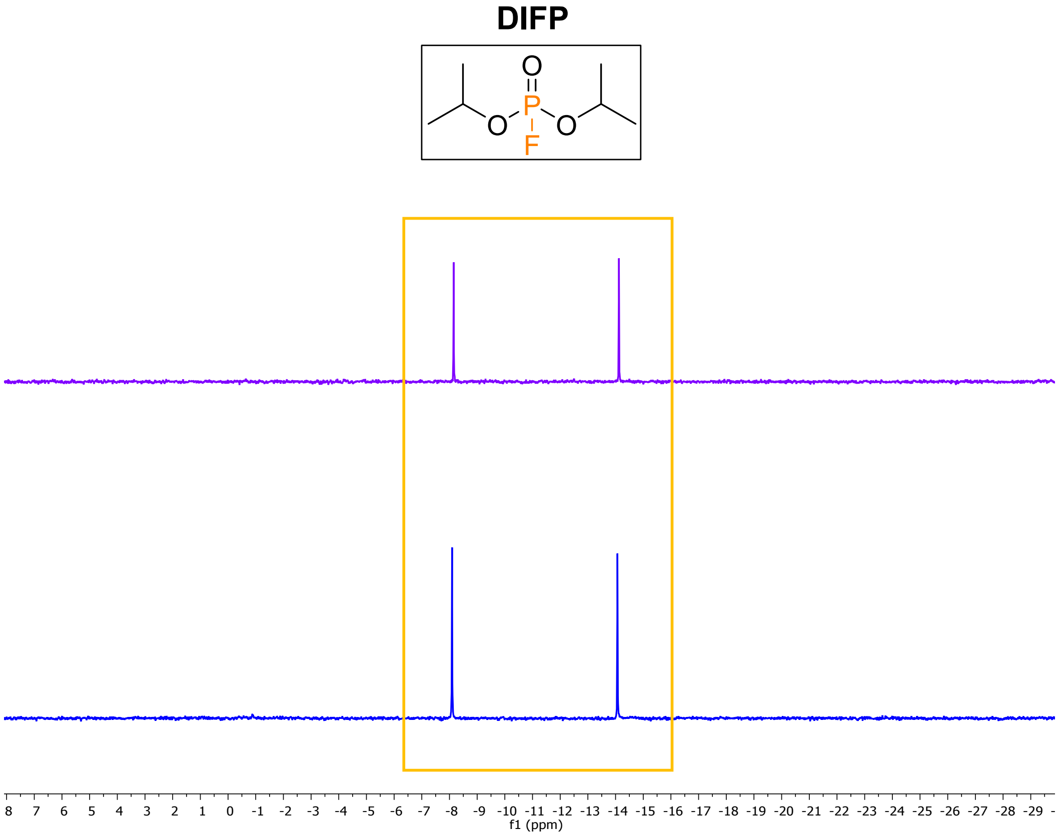

Figure S38. ^{31}P NMR spectra from the study of the adsorption of diisopropylfluorphosphate (DIFP) on **Zr-MOP-1** after 15 minutes (top) and 24 hours (bottom).

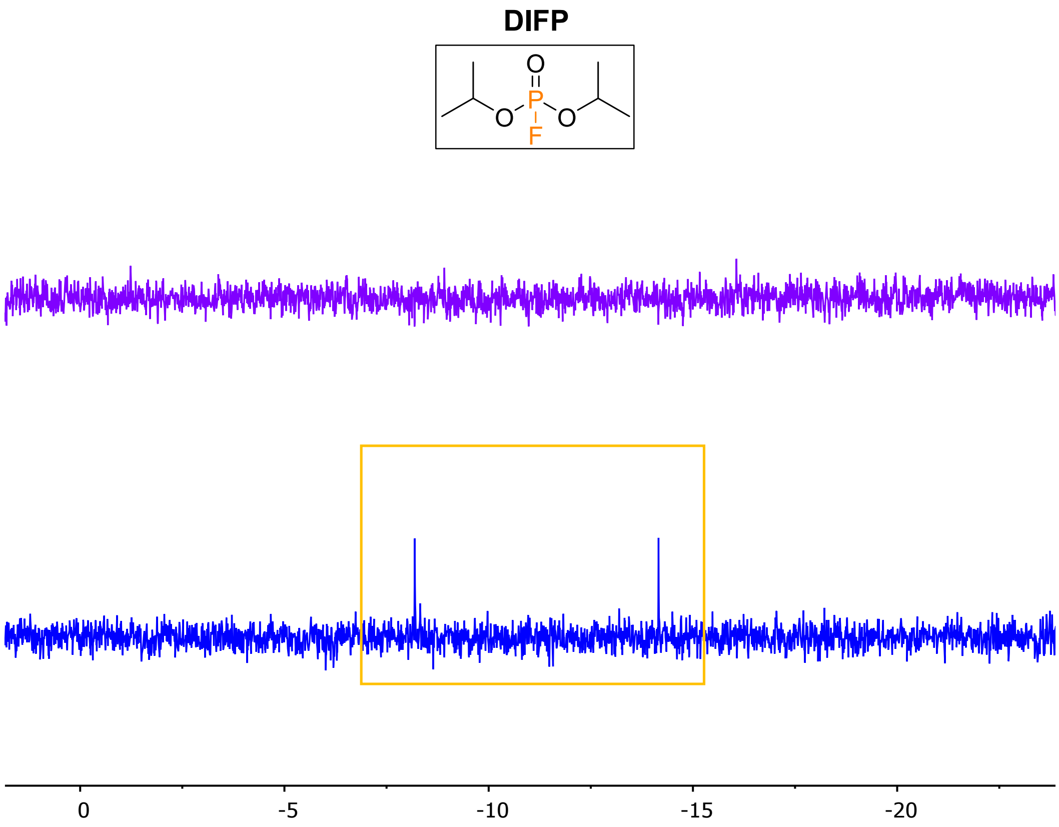

Figure S39. ^{31}P NMR spectra from the study of the adsorption of diisopropylfluorphosphate (DIFP) on **Zr-MOP-1-NH$_2$** after 15 minutes (top) and 24 hours (bottom).

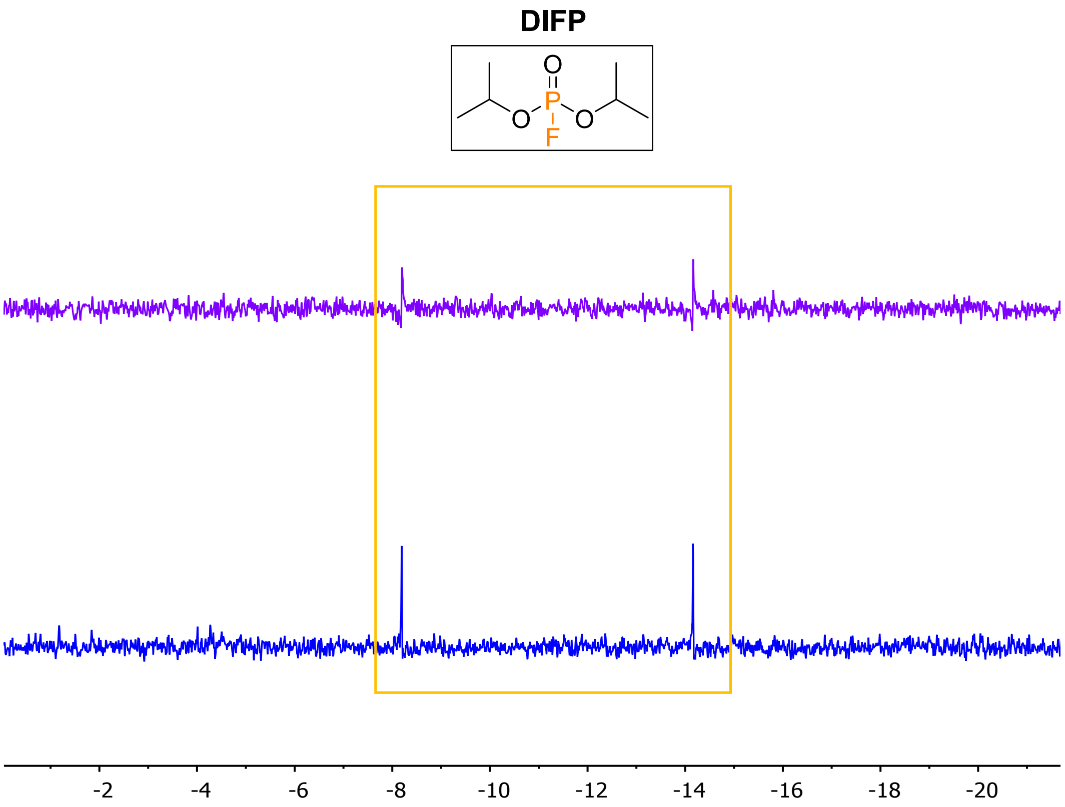

Figure S40. ^{31}P NMR spectra from the study of the adsorption of diisopropylfluorphosphate (DIFP) on **Zr-MOP-10** after 15 minutes (top) and 24 hours (bottom).

S.5. UV-Vis assays.

S.5.1. 2-PAM release from Zr-MOP-1@2-PAM and Zr-MOP-10@2-PAM.

20 mg of **Zr-MOP-1@2-PAM** or **Zr-MOP-10@2-PAM** were suspended in 14.5 mL of Tris-HCl, 4.5 mL of H_2O and 1 mL of isopropanol, to replicate the medium used in activity assays. The mixtures were incubated at 37 °C under orbital shaking. At different time lapses, aliquots of 100 μL were taken and centrifuged. The concentration of 2-PAM in the supernatant was determined by UV-Vis spectroscopy at $\lambda = 293$ nm.

S.5.2. AChE Reactivation Assays.

Activity of AChE was assessed by using the indoxyl acetate-based method with slight modifications.[6] In a typical experiment, 24 well culture plate was filled with 725 Tris-HCl 0.1 M, pH 7.5, and 25 μL of AChE aqueous solution (50 U/mL). Then, 200 μL of water or 200 μL of an aqueous DIFP solution (final concentration 5×10^{-6} M) were added for the control and inhibition study, respectively. The samples were incubated at 37 ºC during 1 h. Afterwards, 50 μL of indoxyl acetate solution in isopropanol (substrate) were added and the mixture was incubated during 30 additional minutes. After this time, each sample was mixed with 3.33 mL of DMSO to solubilize the enzymatic product (indigo blue). Its concentration was determined by UV-Vis spectroscopy at $\lambda = 620$ nm ($\epsilon = 22140$ M^{-1} cm^{-1}) to calculate the enzymatic activity.[7]

In the case of the reactivation studies, 725 mL? of Tris-HCl 0.1 M, pH 7.5, 25 μL of AChE aqueous solution (50 U/L) and 100 μL of an aqueous DIFP solution (final concentration 5×10^{-6} M) were incubated during 30 min at 37 ºC. After this time, 100 μL of a solution of 2-PAM (final concentration of 50 μM) or the supernatant released from **Zr-MOP-1@2-PAM** after 1 or 24 h of incubation were added and the mixture was incubated during 30 additional minutes. Afterwards, 50 μL of indoxyl acetate solution in isopropanol (substrate) were added and the mixture was incubated during 30 additional minutes. After this time, each sample was mixed with 3.33 mL of DMSO to solubilize the enzymatic product (indigo blue). Its concentration was determined by UV-Vis spectroscopy at $\lambda = 620$ nm ($\epsilon = 22140$ M^{-1} cm^{-1}) to calculate the enzymatic activity.[8]

S.6. Computational modelling (Adsorbate locator)

Computational modelling of the interaction of the pralidoxime (2-PAM) drug and the diisopropylfluorophosphate (DIFP) toxic molecules with the **Zr-MOP-1** and **Zr-MOP-10** host frameworks was carried with the BIOVIA Materials Studio 2018 Adsorption Locator module[9] in order to identify the possible adsorption configurations of the studied substrates in the MOP cages by carrying out Monte Carlo searches of the configurational space of the substrate-adsorbate system.

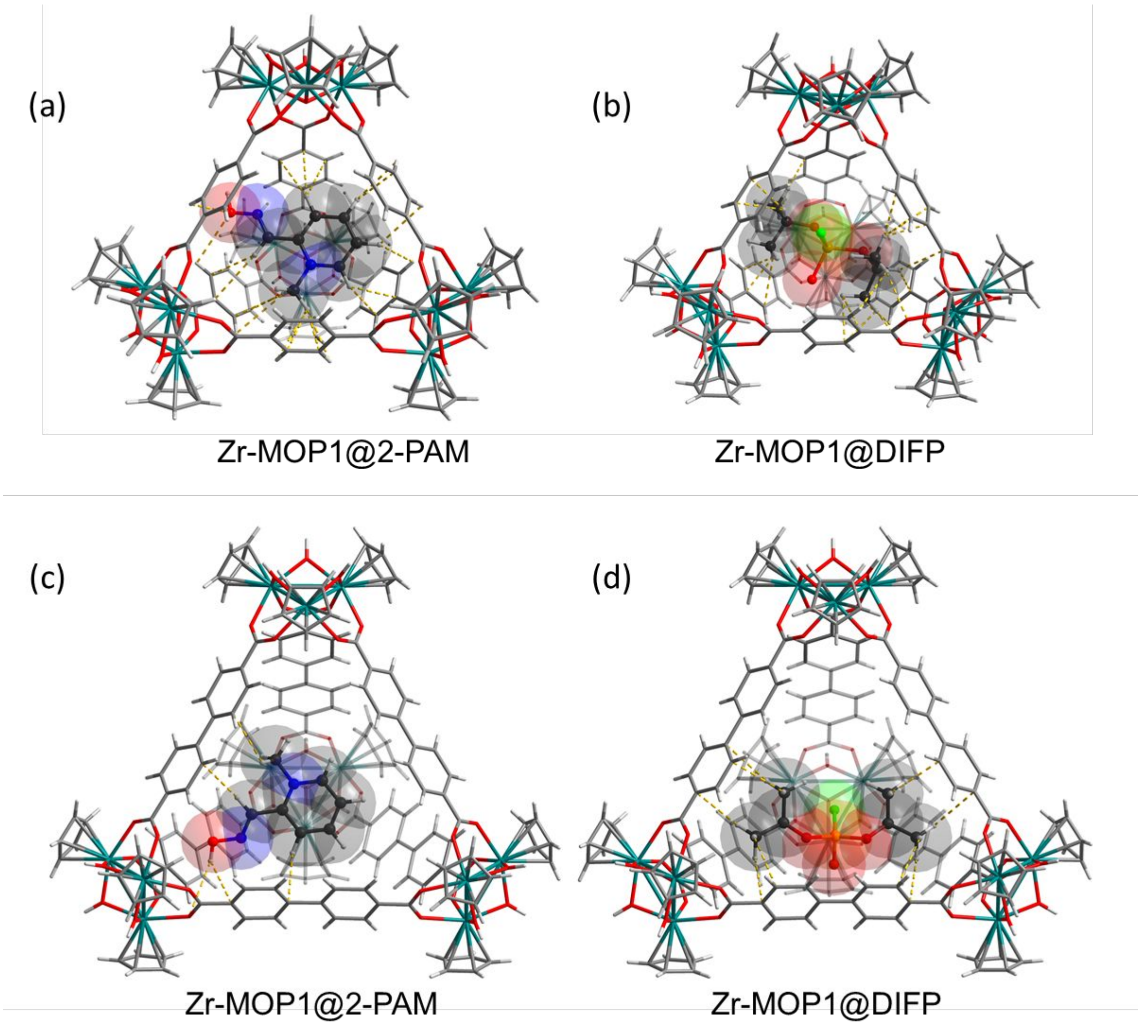

Figure S41. Computational model of the pralidoxime (2-PAM) drug and diisopropylfluorophosphate (DIFP) toxic molecules accommodation in the inner cages of the **Zr-MOP-1** (a, b) and **Zr-MOP-10** (b, c) systems.

S.7. Transmission electron microscopy (TEM)

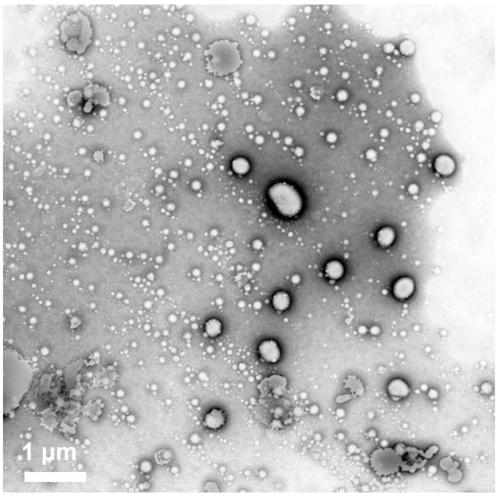

Figure S42. TEM images of **liposome@Zr-MOP-1@2-PAM** assemblies prepared from methanolic solutions of **Zr-MOP-1@2-PAM**.

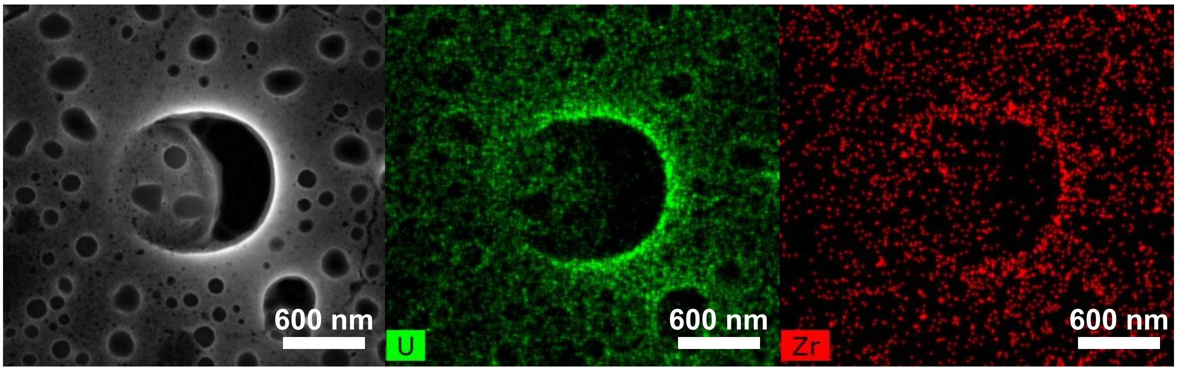

Figure S43. TEM-EDX analysis of **liposome@Zr-MOP-1@2-PAM** assemblies prepared from solutions of **Zr-MOP-1@2-PAM** in $CHCl_3$. (Uranium: Green, Zirconium: Red). Uranium is presented due to the treatment of the assemblies with uranyl acetate.

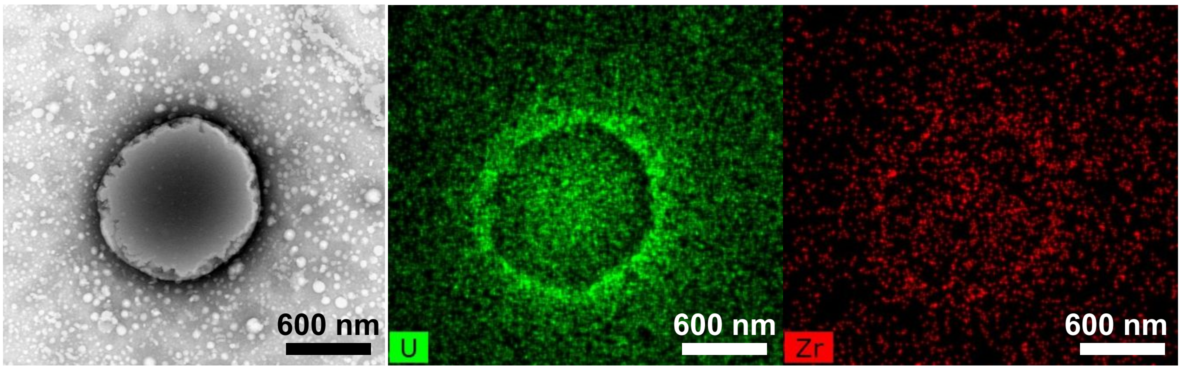

Figure S44. TEM-EDX analysis of **liposome@Zr-MOP-1@2-PAM** assemblies prepared from methanolic solutions of **Zr-MOP-1@2-PAM**. (Uranium: Green, Zirconium: Red). Uranium is presented due to the treatment of the assemblies with uranyl acetate.

References.

1 Lang, C.; Li, W.; Dong, Z.; Zhang, X.; Yang, F.; Yang, B.; Deng, X.; Zhang, C.; Xu, J.; Liu, J. Biomimetic Transmembrane Channels with High Stability and Transporting Efficiency from Helically Folded Macromolecules. *Angew. Chem., Int. Ed.* **2016**, 54, 9723-9727.

2 Blessin, R. H. An empirical correction for absorption anisotropy, *Acta Cryst.* 1995. As1, 33-38

3 Sheldrick, G.M. Crystal structure refinement with SHELXL Acta Cryst. **2015**, *C71*, 3-8

4 Dolomanov, O. V.; Bourhis, L. J.; Gildea, R. J.; Howard, J. A. K.; Puschmann, H. OLEX2: a complete structure solution, refinement and analysis program *J. Appl. Crystallogr.* 2009, 42, 339-341.

5 Spek, A. L. PLATON SQUEEZE: a tool for the calculation of the disordered solvent contribution to the calculated structure factors *Acta Cryst.* **2015**, C71, 9–18.

6 Pohanka, M.; Hrabinova, M.; Kuca, K.; Simonato, J.-P. Assessment of Acetylcholinesterase Activity Using Indoxylacetate and Comparison with the Standard Ellman's Method. *Int. J. Mol. Sci.* **2011**, *12*, 2631-2640.

7 Seixas de Melo, J.; Moura, A. P.; Melo M. J. Photophysical and Spectroscopic Studies of Indigo Derivatives in Their Keto and Leuco Forms *J. Phys. Chem. A* **2004**, *108*, 6975–6981. https://doi.org/10.1021/jp049076y

8 Seixas de Melo, J.; Moura, A. P.; Melo M. J. Photophysical and Spectroscopic Studies of Indigo Derivatives in Their Keto and Leuco Forms *J. Phys. Chem. A* **2004**, *108*, 6975–6981. https://doi.org/10.1021/jp049076y

9 https://www.3ds.com/products-services/biovia/products/molecular-modeling-simulation/biovia-materials-studio/
